# Global proteomics of *Ubqln*2-based murine models of ALS

**DOI:** 10.1074/jbc.RA120.015960

**Published:** 2020-12-10

**Authors:** Alexandra M. Whiteley, Miguel A. Prado, Stefanie A.H. de Poot, Joao A. Paulo, Marissa Ashton, Sara Dominguez, Martin Weber, Hai Ngu, John Szpyt, Mark P. Jedrychowski, Amy Easton, Steven P. Gygi, Thimo Kurz, Mervyn J. Monteiro, Eric J. Brown, Daniel Finley

**Affiliations:** 1 Department of Cell Biology, Harvard Medical School, Boston, Massachusetts, USA; 2 Department of Neuroscience, Genentech Inc, South San Francisco, California, USA; 3 Department of Pathology, Genentech Inc, South San Francisco, California, USA; 4 Henry Wellcome Lab of Cell Biology, College of Medical, Veterinary and Life Sciences, Institute of Molecular, Cell and Systems Biology, University of Glasgow, Glasgow, United Kingdom; 5 Center for Biomedical Engineering and Technology, Department of Anatomy and Neurobiology, University of Maryland Medical School, Baltimore, Maryland, USA; 6 Department of Immunology and Infectious Diseases, Genentech Inc, South San Francisco, California, USA

**Keywords:** ubiquitin, proteasome, ALS, neurodegeneration, proteomics, ubiquitin ligase, UBQLN2, AA, active avoidance, ACN, acetonitrile, AHA, azido-homo-alanine, ALS, amyotrophic lateral sclerosis, BCA, bicinchoninic acid assay, CFP, cyan fluorescent protein, fALS, familial genetic variants of ALS, FC, fear conditioning, GO, gene ontology, iNeuron, induced neuron, ITI, intertrial interval, KI, knock-in, qPCR, quantitative PCR, RFP^+^, red fluorescent protein positive, TKO, triple knockout, TMT, tandem mass tag, UBA, ubiquitin-binding domain, UBL, ubiquitin-like, UBQLN, ubiquilin proteins, UCS, unconditioned stimuli

## Abstract

Familial neurodegenerative diseases commonly involve mutations that result in either aberrant proteins or dysfunctional components of the proteolytic machinery that act on aberrant proteins. UBQLN2 is a ubiquitin receptor of the UBL/UBA family that binds the proteasome through its ubiquitin-like domain and is thought to deliver ubiquitinated proteins to proteasomes for degradation. *UBQLN2* mutations result in familial amyotrophic lateral sclerosis (ALS)/frontotemporal dementia in humans through an unknown mechanism. Quantitative multiplexed proteomics was used to provide for the first time an unbiased and global analysis of the role of *Ubqln2* in controlling the composition of the proteome. We studied several murine models of *Ubqln2*-linked ALS and also generated *Ubqln2* null mutant mice. We identified impacts of *Ubqln2* on diverse physiological pathways, most notably serotonergic signaling. Interestingly, we observed an upregulation of proteasome subunits, suggesting a compensatory response to diminished proteasome output. Among the specific proteins whose abundance is linked to UBQLN2 function, the strongest hits were the ubiquitin ligase TRIM32 and two retroelement-derived proteins, PEG10 and CXX1B. Cycloheximide chase studies using induced human neurons and HEK293 cells suggested that PEG10 and TRIM32 are direct clients. Although UBQLN2 directs the degradation of multiple proteins *via* the proteasome, it surprisingly conferred strong protection from degradation on the Gag-like protein CXX1B, which is expressed from the same family of retroelement genes as PEG10. In summary, this study charts the proteomic landscape of ALS-related *Ubqln2* mutants and identifies candidate client proteins that are altered *in vivo* in disease models and whose degradation is promoted by UBQLN2.

Amyotrophic lateral sclerosis (ALS) is a progressive and fatal motor neuron disease that typically presents in the fourth to seventh decades of life and affects upward of 30,000 adults in the United States (1). Like many progressive neurodegenerative diseases, ALS features an accumulation of protein aggregates that are thought to promote or cause disease through the progressive loss of neuronal function (2, 3). Familial genetic variants of ALS (fALS) often lead to misfolding of the mutant protein, which can be toxic and drive the development of disease, as suggested for TDP-43, SOD1, and C9ORF72 mutations (4). In other cases, mutations in components of the protein degradation machinery itself result in the accumulation of aggregates and other disease manifestations (5, 6). Examples, for fALS, include optineurin, p62/SQSTM1, VCP, and ubiquilin2 (UBQLN2; [7]).

UBQLN2 belongs to a large family of ubiquilin proteins (UBQLNs) that link proteasomes with ubiquitinated proteins through a ubiquitin-like domain (UBL) that binds to the proteasome and a ubiquitin-binding domain (UBA) that binds to a variety of mono- or polyubiquitinated proteins (8, 9, 10, 11). As such, UBQLNs and other UBL/UBA proteins facilitate proteasomal degradation of ubiquitinated proteins. Our understanding of Ubqln biology is largely based on studies of the yeast homolog Dsk2 (12, 13, 14, 15), the UBL domain of which is recognized by subunits Rpn10, Rpn13, and Rpn1 of the proteasome (16, 17, 18, 19). Mammalian UBQLNs appear to bind the proteasome *via* the orthologous subunits (8, 18, 20). The UBA domain of Dsk2 and mammalian UBQLNs binds ubiquitin chains (21, 22, 23) but has little or no inherent preference for particular ubiquitin linkages (11, 24, 25, 26, 27, 28). However, for some clients, UBQLNs may bind their nonubiquitinated forms and instead promote ubiquitination through recruitment of a ligase (29, 30).

Although Dsk2 appears to promote the degradation of a large fraction of ubiquitinated proteins (22, 24, 31), UBQLNs have been implicated in the degradation of specific classes of proteins, most strikingly hydrophobic and/or transmembrane proteins (32), aggregates (33), and mislocalized mitochondrial proteins (30, 34). Although these reports implicate different potential functions of UBQLNs, some of their apparent functional diversity may be due to the multiplicity of Ubqln isoforms. There are six *UBQLN* genes expressed in humans: *UBQLN1*, which is expressed throughout the body; *UBQLN2*, which is enriched in neuronal tissues and muscle; *UBQLN 3*, *5*, and *L*, which are expressed only in testes; and *UBQLN4*, which is induced in many cell types upon nutrient limitation (35). Although all *UBQLN* genes have a similar domain organization featuring UBL and UBA domains, many also contain unique motifs that could potentially confer client specificity or functional specialization. For example, UBQLN4 possesses a noncanonical LC3-binding motif (36), which is thought to confer the ability to promote autophagic protein degradation (37, 38, 39, 40), and UBQLN2 has also been implicated in the control of autophagy (41). Notably, UBQLN2 contains a proline-rich repeat (the PXX domain), which may modulate client specificity (42), proteasome binding (43), and liquid–liquid phase separation into stress granules (29, 44, 45, 46).

Mutations in *UBQLN2* cause a heritable, X-linked, dominant form of ALS with frontotemporal dementia in humans (47). This discovery has been corroborated through the use of mutant transgenic mouse and rat models, some of which mimic *in vivo* symptoms of motor neuron disease and exhibit protein aggregates in the brain (33, 43, 46, 48, 49). Although the initially identified mutations were all located in the PXX domain, recent studies have also identified patients with mutations adjacent to or distant from this region (47, 50, 51, 52, 53). The molecular mechanism of neurodegenerative disease due to the *UBQLN2* mutation remains unknown but could involve progressive accumulation of as-yet-unidentified Ubqln target proteins. However, there have been no published comprehensive proteomic studies in any relevant model system to identify target proteins in an unbiased fashion.

UBQLNs have been shown to regulate the abundance of individual proteins linked to neurodegenerative disease, including Huntingtin (33, 48, 54, 55), Aß (56), and presenilins (57). Furthermore, UBQLN2 colocalizes with TDP-43 inclusions of fALS not only when UBQLN2 is mutated (47) but also in fALS characterized by an ALS-associated FUS mutation (51) or mutant TDP-43 overexpression (47). Like other UBQLNs, UBQLN2 oligomerizes (58) and is capable of phase separation, which could potentially contribute to disease by influencing protein degradation pathways and even nucleating protein aggregation within the cell (29, 44, 45, 46). Thus, perturbation of UBQLN2 function may promote neurodegeneration by compromising the proteasomal degradation of proteins known to cause ALS, such as TDP-43. Alternatively, *Ubqln2* mutations may lead to neurodegeneration through the dysregulation of unrelated, unknown proteins, through a general attenuation of protein quality control, or through an inherent toxicity that is unrelated to the normal function of UBQLN2 in protein degradation.

In this study, we have performed a comprehensive proteomic analysis of multiple animal models of *Ubqln2*-driven neurodegenerative disease. In doing so, we have identified proteins whose abundance changes upon *Ubqln2* perturbation and thus are not only linked to Ubqln2 function but could also potentially be involved in the development of neurodegenerative disease. Furthermore, through the utilization of pathway analysis we were also able to identify groups of proteins that were functionally linked to *Ubqln2*. By comparing results from multiple *Ubqln2*-dependent models of ALS, including both young and old animals, we were able to focus our analysis on a select group of proteins that were commonly altered upon different forms of *Ubqln2* perturbation and to assess their relationship to UBQLN2. From this global search we have identified exceptionally responsive UBQLN2 clients in the brain, notably the E3 ubiquitin ligase TRIM32 and the retroelement product PEG10. Unexpectedly, we also found UBQLN2 to strongly protect from degradation the Gag-like protein CXX1B, which is expressed from the same family of retroelement genes as PEG10–the Mar family. In conclusion, our results elucidate the proteomic changes that occur throughout the disease course of Ubqln2-mediated neurodegeneration in murine model systems, revealing potential pathways of disease progression as well as novel putative clients of UBQLN2.

## Results

### *Ubqln2*^*−/−*^ animals develop age-dependent neuromotor defects

**Figure 1:**
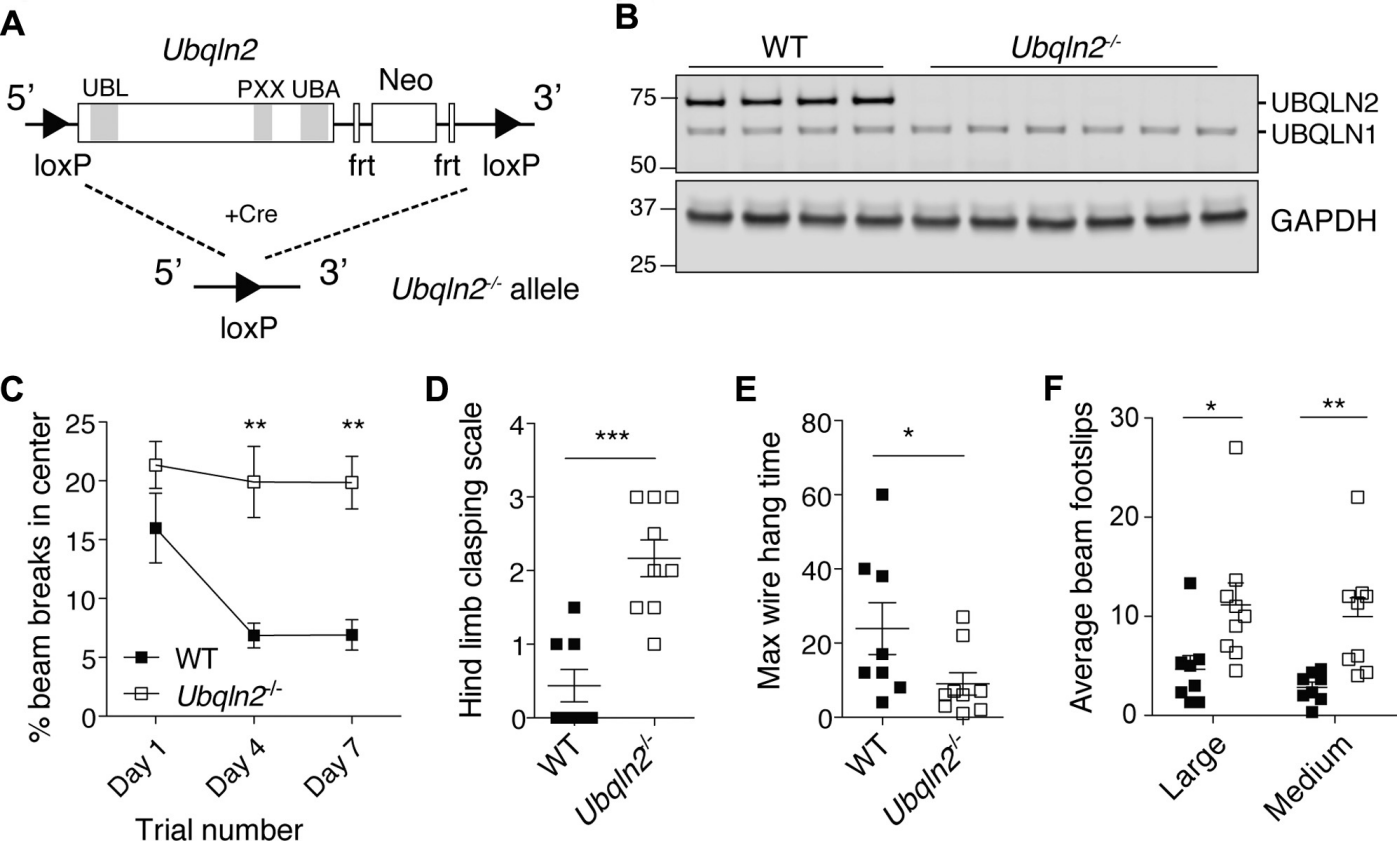
**Neurodegenerative phenotype of *Ubqln2***^***−/−***^**mice**. *A*, schematic representation of *Ubqln2* deletion. Functional domains are annotated and shown in *gray*. *B*, western blot demonstrating loss of UBQLN2 protein, but not UBQLN1, in spinal cord samples of 5- to 6-month-old mice. Numbers at left indicate location of molecular weight markers in kilodaltons. *C–F*, age-matched 10- to 14-month-old male mice were tested in neuromotor exams. *C*, *Ubqln2*^*−/−*^ mice were more active over multiple sessions in an open field test as measured by percentage center beam breaks in the course of three sessions. Statistical significance was determined with repeated measures ANOVA. *D* and *E*, *Ubqln2*^*−/−*^ mice exhibit increased hind limb clasping upon tail suspension and (*E*) decreased wire hang time compared with littermates. *F*, *Ubqln2*^*−/−*^ mice have increased footslips when on large and medium balance beam. n = 8 to 9 mice per genotype. Statistical significance for (*D–F*) was determined with unpaired, two-tailed Student's *t* test. Frt, Flp recombination sites; Neo, neomycin resistance cassette.

*Ubqln2*-deficient mice were generated by flanking the one exon of murine *Ubqln2* with *loxP* sites (Fig. 1*A*) and expressing Cre-bearing plasmids in flox-bearing C57BL/6 ES cells. Complete loss of the UBQLN2 protein was confirmed by western blot of spinal cord tissue from WT and *Ubqln2*^*−/−*^ animals (Fig. 1*B*), whereas UBQLN1 protein levels were unaffected by the loss of UBQLN2 (Fig. 1*B*). At weaning, there were no gross motor defects in *Ubqln2*^*−/−*^ pups compared with their WT littermates. However, when older animals (10- to 14-month-old males) were tested in a battery of neuromotor exams, the *Ubqln2*^*−/−*^ mutants consistently displayed a pleiotropic phenotype. In open field tests they exhibited more center beam breaks (Fig. 1*C*; see also Table S1), indicating hyperactivity, or deficient anxiety, during repeated testing. *Ubqln2*^*−/−*^ mice also exhibited increased hind limb clasping (Fig. 1*D*), decreased wire hang time (Fig. 1*E*), and increased footslips on both large and medium balance beams (Fig. 1*F*) as compared with WT littermates, despite no differences in weight (Table S1). Aged *Ubqln2*^*−/−*^ mutants demonstrated transient changes in active avoidance of aversive stimuli (Fig. S1, *A*–*B*) and mild defects in fear conditioning (Fig. S1, *C*–*E*). Despite these phenotypes, there were no detectable changes in the number of neurons, markers of neuroinflammation, or ubiquitin-containing aggregates in the *Ubqln2*^*−/−*^ spinal cord (Fig. S2; note that ALS-associated aggregates are typically ubiquitin positive [1]). From these results, we concluded that these animals have an intermediate, age-dependent neuromotor disease more severe than that seen in *Ubqln2* knock-in mice containing a mutant murine P520T *Ubqln2* allele corresponding to the human disease-causing P506T mutation (33) but less severe than that of mice overexpressing a disease-causing mutant human P497S *UBQLN2* allele (59).

### *Ubqln2*^*−/−*^ tissues demonstrate proteomic changes prior to the development of neuromotor defects

**Figure 2:**
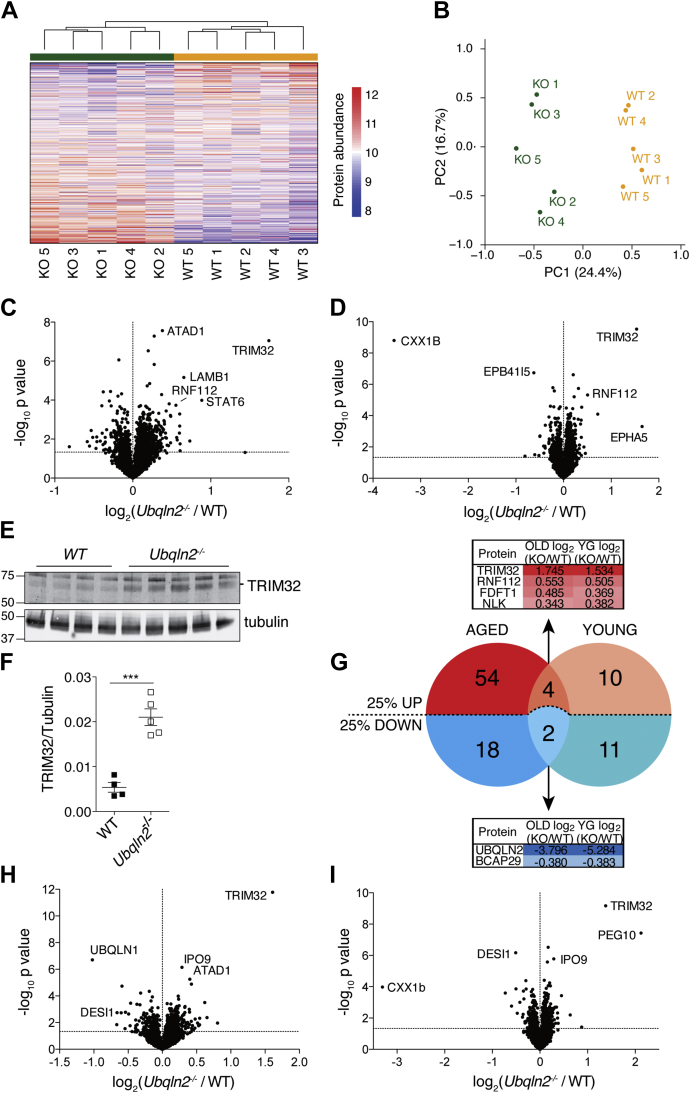
**Proteomics of brain and spinal cord reveals proteins changed upon *Ubqln2* loss**. *A–C*, brain hemispheres from 12- to 16-month-old WT (n = 5) and *Ubqln2*^*−/−*^ (KO, n = 5) mice were isolated and their proteome analyzed by tandem mass tag (TMT). *A*, heat map with unsupervised hierarchical clustering analysis using Euclidean distance of WT (*yellow*) and KO (*green*) brain hemisphere samples. *B*, principal component analysis of WT (*yellow*) and KO (*green*) brain samples. *C*, volcano plots with log_2_ ratios of mean protein abundances of all quantified proteins, with increasing significance on the *y*-axis (*p*-value of 0.05 noted with a *horizontal dashed line*). *D*, brain hemispheres were dissected from younger mice (5–6 months old) for TMT analysis. n = 6 Ubqln2^−^^/^^−^, and n = 4 WT mice in both experiments. *E*, spinal cord of 5- to 6-month-old animals was lysed in urea buffer and prepared for western blotting of TRIM32 and tubulin. *F*, TRIM32 quantification of (*E*) normalized to tubulin. Statistical significance was determined with two-tailed, unpaired Student's *t* test. *G*, Venn diagram of proteins from aged (*C*) and young (*D*) brain hemispheres that are up- or downregulated >25% (*p*-value < 0.05). Four proteins were increased at least 25% in both aged (OLD) and young (YG) tissues (top, *red*) and two proteins were decreased at least 25% in both (bottom, *blue*). Tables highlight the log_2_ ratios of each named protein from Table S2. *H* and *I*, Hippocampus (n = 5 samples from three biological replicates, for both genotypes) was isolated from 5- to 6-month-old mice and (*I*) lumbar spinal cord (n = 6 *Ubqln2*^*−/−*^ and 4 WT) was isolated from 4-month-old animals for TMT analysis. Information on the total number of proteins identified and quantified in each TMT from this study is included in Tables S2 and S3.

Our main interest in *Ubqln2*^*−/−*^ animals was to examine neural tissue for possible changes to the proteome. Therefore, five unique brain hemispheres of each genotype were isolated from mice at 12 to 16 months of age for proteomic analysis (Table S1). Homogenized protein samples were digested and labeled with tandem mass tags (TMTs) for multiplexed quantitative proteomic analysis using SPS-MS3. Both hierarchical clustering (Fig. 2*A*) and principal component analysis (Fig. 2*B*) of proteomics data demonstrated distinct separation of samples based on the *Ubqln2* genotype. Changes to individual proteins are highlighted in Figure 2*C*; there was a substantial upregulation of the ubiquitin ligases TRIM32 and RNF112 (also known as neurolastin or ZNF179), both of which are known to regulate neuronal development and function (60, 61). Mutations within the TRIM32 NHL domains cause the neuromuscular disease Limb-Girdle muscular dystrophy type 2H, whereas those within its BBOX domain result in the ciliopathic disorder Bardet-Biedl syndrome (62, 63). TRIM32 ubiquitinates dysbindin (64) and actin (65), *via* K48 ubiquitin linkages, and MITA/STING (66) *via* K63 linkages, whereas RNF112/ZNF179 has been found to ubiquitinate TDP-43 and target it for degradation (67). Finally, there was an increase in the mitochondrial protein ATAD1 that, although lesser in magnitude, was highly significant statistically (Fig. 2*C*). ATAD1 and its yeast homolog Msp1 remove mislocalized tail-anchored proteins from the mitochondrial membrane (68, 69); ATAD1 upregulation has also been observed upon loss of *Ubqln1* (34), which facilitates degradation of mislocalized mitochondrial proteins (30). Thus, the upregulation of ATAD1 in *Ubqln2*-deficient tissues implicates a shared pathway linking UBQLN1, UBQLN2, mislocalized mitochondrial proteins, and ATAD1 protein abundance.

Because samples were taken for proteomics from older animals that exhibited *in vivo* neuromotor defects, changes to the proteome could reflect chronic disease as opposed to an acute disturbance due to *Ubqln2* loss. We were particularly interested in observing the early proteomic changes that predate overt phenotypic effects of *Ubqln2* loss. Therefore, proteomics was also performed on brain hemispheres of animals at 5 to 6 months of age, when tests that track motor neuron dysfunction did not show statistically significant differences between mutant and WT (Fig. S3). A small number of proteins were dramatically changed in *Ubqln2*^*−/−*^ hemispheres of young mice, many of which were shared with older brain samples. As in aged animals, the ubiquitin ligases TRIM32 and RNF112 were upregulated, particularly TRIM32 (Fig. 2*D*), which was also confirmed by western blotting (Fig. 2, *E*–*F*). Although most affected proteins were upregulated in *Ubqln2*^*−/−*^ tissues compared with their WT counterparts, in *Ubqln2*^*−/−*^ brains there was a dramatic loss of the protein CXX1B, which was likewise confirmed by western blotting (Fig. S4*A*). CXX1B, also known as FAM127 or RTL8, is a Gag-like protein (Fig. S4) of unknown function expressed from specific members of the Ty3/Gypsy family of endogenous LTR-derived retroelements (70).

Proteomic hits from brain hemisphere samples could be divided into three groups: those that were similarly affected by *Ubqln2* loss regardless of age, as expected of UBQLN2 client proteins; those showing more dramatic changes in aged *Ubqln2*^*−/−*^ animals; and those altered in only young but not aged *Ubqln2*^*−/−*^ animals (Fig. 2*G*). The E3 ligases TRIM32 and RNF112, as well as the metabolic regulator FDFT1 and Nemo-like kinase, did not significantly change upon aging of *Ubqln2*^*−/−*^ animals, indicating that these are early and persistent changes to the proteome upon *Ubqln2* loss. In addition, the B cell receptor–associated protein 29 was downregulated in both aged and young brains (Fig. 2*G*). LAMB1 and STAT6 were only significantly upregulated in aged tissues (Fig. 2*C*) and appear to represent sensitive indicators of the progression of neuromotor disease in *Ubqln2*^*−/−*^ animals. EPHA5 and EPB41L5, on the other hand, were both altered in young tissues and unaffected by *Ubqln2* loss in aged animals.

To further define the effects of Ubqln2 on tissue proteomes relevant to the development of ALS, we isolated regions of CNS tissue known to express high levels of UBQLN2 and to be affected in ALS. Hippocampus and lumbar spinal cord were isolated from *Ubqln2*^*−/−*^ mice between 4 and 6 months of age (Table S1), before they exhibited significant motor defects (Fig. S3), and tissue was prepared for proteomics (Fig. 2, *H*–*I*). Most notably, TRIM32 was found to be elevated in these tissues as a result of the *Ubqln2* null mutation, similarly to the brain hemisphere samples. ATAD1 was elevated upon *Ubqln2* loss in the hippocampus as had been observed in brain hemisphere samples. Unlike the spinal cord and brain, hippocampus showed a clear decrease in UBQLN1 protein expression (Fig. 2*H*), which may reflect a difference among tissues in their response to *Ubqln2* loss. In the case of spinal cord, there was also a dramatic loss of CXX1B, comparable with that described above for young brain. In addition, spinal cord revealed a strong upregulation of the protein PEG10, which, like CXX1B, is expressed from specific members of the Ty3/Gypsy family of retroelements (70).

PEG10 is expressed primarily in two forms, PEG10-RF1 and PEG10-RF1/2, which correspond to the retroviral gene products Gag and Gag-Pol, respectively (Fig. S4, *B*–*C*). In retroviruses, the Pol element encodes a retroviral protease, reverse transcriptase, and integrase, whereas PEG10-RF1/2 lacks reverse transcriptase and integrase activities owing to deletions within this element. PEG10-RF1/2 is, like the ancestral Gag-Pol protein, generated by a highly efficient translational frameshift between the Gag-like and Pol-like elements (71, 72, 73, 74). Although in retroviruses the Pol element is subsequently cleaved from the Gag element, this proteolytic cleavage is at best weak in PEG10 (75). Instead, the “Gag-only” form PEG10-RF1 is produced when the frameshift element is bypassed and the proximal stop codon is engaged (Fig. S4*C*).

PEG10 upregulation promotes the proliferation and invasiveness of cancer cells (76), and deletion in mouse models causes lethality owing to placentation defects (77). Recently, it was found that PEG10 regulates mRNA abundance in murine trophoblast cells, thereby promoting their differentiation in culture (75). Furthermore, purification of PEG10-RF1 yielded virus-like particles (75), similar to the Gag-like protein ARC/ARG3.1, which binds RNAs and facilitates intercellular RNA transport among neurons (78, 79). Of interest, the abundance of PEG10-RF1, but not RF1/2, was found to be regulated by the deubiquitinating enzyme USP9X, which specifically protects the short form against proteasomal degradation (75).

CXX1B is expressed from members of the same Ty3/Gypsy retroelement family as PEG10 and is similar to PEG10-RF1 in being composed almost entirely of a Gag-like domain (Fig. S4*C*). CXX1B has significant sequence homology to the Gag-like domain of PEG10, as well as the ancestral Gag region of the Sushi-ichi retroelement, although it has little homology to the ancestral Gag region of retroviruses and is lacking the CCHC domain of PEG10 and other Gag-like regions (Fig. S4*D*).

Expression of TRIM32, PEG10, and CXX1B is enriched in WT murine CNS tissues compared with many other tissue types. At the mRNA level, both *Ubqln2* and *Trim32* show high levels of expression in a variety of neuronal subtypes (Fig. S5, *A*–*B*). *Cxx1b* mRNA is highly expressed in a variety of neuronal subtypes and oligodendrocytes (Fig. S5*C*). *Peg10* mRNA is most highly enriched in transitional tissues present during development, followed by oligodendrocytes and placental cell types (Fig. S5*D*). In contrast to the related *Peg10*, however, TMT analysis of multiple murine tissues found enrichment of both UBQLN2 and TRIM32 proteins in brains (Fig. S6*A*, top), and a similar label-free analysis found PEG10 enrichment in testis and CXX1B enrichment in brain and lung (Fig. S6*A*, bottom). Additional studies have observed high levels of PEG10 protein in CNS tissue (73, 75). CXX1B, TRIM32, and UBQLN2 proteins were all enriched at least twofold in brain tissue as compared with liver (Fig. S6*B*).

### Ubiquilin dependence of PEG10, TRIM32, and CXX1B abundance is conserved between mouse and humans

**Figure 3:**
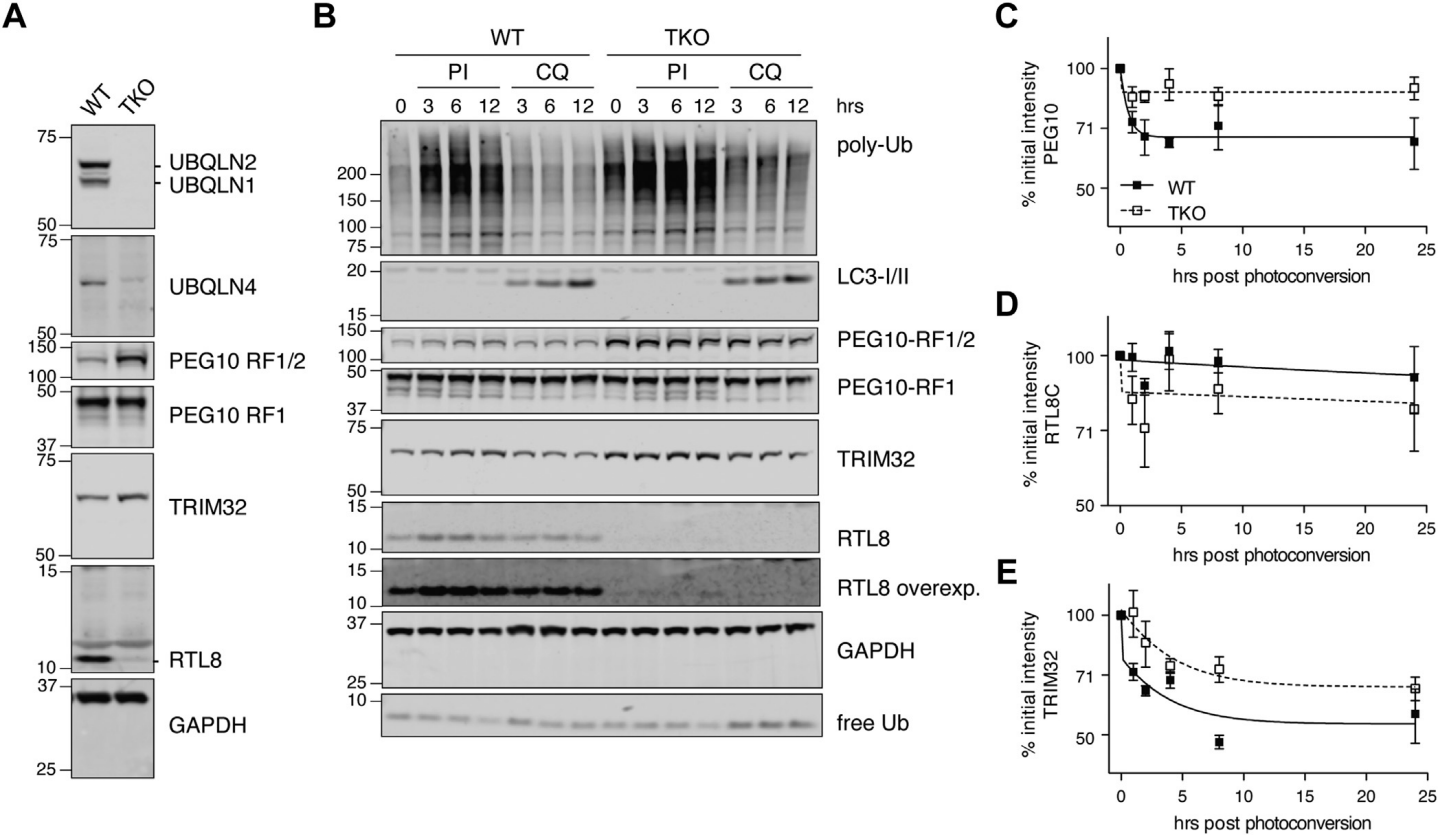
**Validation and proteasome dependence of top hits in HEK 293 cells**. *A*, western blot demonstrating similar changes in TRIM32, PEG10, and RTL8 protein levels in HEK 293 cells lacking UBQLN1, 2, and 4 (labeled TKO). *B*, WT or triple knockout (TKO) HEK 293 cells were treated with bortezomib plus epoxomicin (50 nM each) or chloroquine (50 μM), for 0, 3, 6, or 12 h to inhibit proteasomal or lysosomal degradation, respectively. Inhibition of degradation was confirmed with either poly-ubiquitin (Ub) accumulation and free ubiquitin (Ub) depletion or LC3-I/II conversion, respectively. Western blots were probed for top UBQLN2 hits. *C–E*, WT or TKO HEK 293 cells were transfected with constructs bearing C-terminal fusions of (*C*) PEG10-RF1/2, (*D*) RTL8C, and (*E*) TRIM32 with Dendra2 fluorophore with an IRES-CFP transfection control. After 48 h, cells were exposed to 60 s of intense blue light to induce photoconversion of Dendra2 and samples were taken at the indicated timepoints for quantitation of the RFP/CFP ratio via flow cytometry. Lines of best fit to biphasic decay were plotted in Prism for each time course. n = 4 independent experiments for each construct. RTL8 overexp., overexposure of RTL8 blot.

To investigate the mechanism of protein accumulation under *Ubqln*-deficient conditions, and its relevance to humans, a cell culture system was used. WT or *UBQLN1*, *2*, and *4* triple-knockout (TKO) HEK 293 cells (30), which are of human origin, were examined for expression of top proteomic hits from Figure 2. Cells lacking Ubqlns exhibited an accumulation of PEG10-RF1/2 but not of PEG10-RF1, as well as accumulation of TRIM32 (Fig. 3*A*). TKO cells also lacked appreciable expression of RTL8, the human ortholog of CXX1B (Fig. 3*A*). In humans, CXX1B homologs are expressed from three loci and are indistinguishable by western blotting: RTL8A (also known as FAM127B), RTL8B (also known as FAM127C), and RTL8C (known as FAM127A), although murine CXX1B has the highest sequence homology to RTL8C. mRNA levels of each protein were compared between WT and TKO cells. *TRIM32* was unchanged by Ubqln loss, whereas *PEG10* showed modest changes in mRNA expression that cannot account for the observed changes at the protein level (Fig. S7*A*). In addition, *RTL8* gene expression was upregulated upon Ubqln loss despite a near-total loss of the protein (Fig. S7*A*). We generally observed similar mRNA levels of *Trim32*, *Peg10*, and *Cxx1b* in WT *versus Ubqln2*^*−/−*^ murine neuronal tissues (Fig. S7, *B*–*D*), indicating that the changes in the level of these proteins were not due to mRNA expression level changes, although there appears to be at least a partial contribution at the RNA level for PEG10 in spinal cord.

In addition to examining HEK 293 cells, we generated human ES cells lacking *Ubqln2* and infected with lentivirus expressing Ngn2 under doxycycline control (80) to induce neural differentiation. Induced neurons (iNeurons) were tested for protein expression of TRIM32, PEG10, and RTL8 (Fig. S8). TRIM32 levels were unaltered by *UBQLN2* loss in iNeurons, unlike in mouse tissues (Fig. 2) and human TKO HEK 293 cells (Fig. 3). The PEG10-RF1/2 protein was upregulated between 2- and 4-fold in *Ubqln2*^*−/−*^ iNeurons, whereas PEG10-RF1 was unaffected by *Ubqln2* loss (Fig. S8, *A*–*B*). The RTL8 protein as measured by western blotting was often present at very low levels in *Ubqln2*^*−/−*^ iNeurons (Fig. S8, *A*–*B*), confirming results from mouse and TKO HEK 293 cells. Quantitative PCR (qPCR) was performed on mRNA from iNeurons to determine whether these proteins were also regulated at the transcriptional level. mRNA levels were not significantly changed in *Ubqln2*^*−/−*^ iNeurons compared with WT controls (Fig. S8*C*). In conclusion, several hits from the *Ubqln2*^*−/−*^ animal studies and TKO HEK 293 cells, including RTL8 and PEG10 RF1/2, are also dysregulated in human iNeurons lacking functional UBQLN2.

To determine whether UBQLN2 has potential clients or interacting partners in addition to those showing altered levels in UBQLN2 KO, knock-in (KI), or transgenic mouse tissues, we performed proximity-dependent biotinylation in HEK 293 TKO cells using a modified plant peroxidase tagged with V5 (V5-APEX2) and fused to the N terminus of UBQLN1 or UBQLN2. On comparison of proteins differentially modified by these constructs, we identified 211 proteins with a 1.5-fold or greater enrichment (and an adjusted *p*-value < 0.05) in APEX2-UBQLN2 samples as compared with those of UBQLN1 (Fig. S9 and Supplemental Discussion). Gene ontology (GO) and Kyoto Encyclopedia of Genes and Genomes pathway analyses identified an enrichment in APEX2-UBQLN2 samples of several processes related to the endocytic machinery such as endosomal transport, multivesicular body assembly, and the endosomal sorting complex required for transport pathway. These analyses also identified regulators of receptors, such as the tumor necrosis factor, and receptor tyrosine kinases, including the epidermal growth factor receptor. These results agree with (81), which reported that UBQLN2 is important for the endocytosis of G protein–coupled receptors and acts as a specific interactor of the epidermal growth factor receptor substrate 15 (EPS15) and epsins (EPN1 and EPN2), all of which are implicated in clathrin-mediated endocytosis (82) and also enriched in our APEX2-UBQLN2 samples. Interestingly, in all these processes, K63 ubiquitin linkages play a critical role (83, 84, 85). In our APEX2 experiment, we also noticed an accumulation of ubiquitin K63 linkages, among others, an observation that agrees with recent findings that full-length UBQLN1 binds preferentially to K63-linked ubiquitin chains in comparison with K48 chains (25); full-length UBQLN2 has not been tested yet. Moreover, APEX2-UBQLN2 enriched for 14 ubiquitin ligases and deubiquitinating enzymes, and several ubiquitin receptors, such as SQTSM1 and NBR1, that are all known to have functions related to K63 linkages. Finally, the APEX2 data were consistent with the assignment of RTL8B, TRIM32, and PEG10 as client proteins of UBQLN2. The APEX2 results suggest that UBQLNs may not only deliver proteins to the proteasome to be degraded but also promote proteasome-independent signaling roles of ubiquitin. For more information regarding this experiment, see Supplemental Discussion and Table S4 (86, 87, 88, 89, 90, 91, 92, 93, 94, 95, 96, 97, 98, 99, 100, 101, 102, 103, 104, 105, 106, 107, 108, 109, 110).

### Proteasome dependence of *Ubqln2* effects

The simplest hypothesis for the accumulation of PEG10 and TRIM32 in *Ubqln2*^*−/−*^ tissues and cells is an impairment of their proteasomal degradation. In support of this hypothesis, ubiquitin-conjugated forms of TRIM32, PEG10, and CXX1B/RTL8 have all previously been observed to accumulate in HCT116 cells treated with proteasome inhibitors (Fig. S10*A*) (111). To examine their turnover in the context of *Ubqln* deficiency, WT and TKO HEK 293 cells were treated with proteasome inhibitors (bortezomib plus epoxomicin) or an autophagy inhibitor (chloroquine). Inhibition of proteasomal degradation was confirmed by observing elevated levels of ubiquitin-conjugates (Fig. 3*B*); after 6 h of treatment, WT cells had accumulated substantial amounts of PEG10-RF1/2 and TRIM32 (Fig. 3*B*). TKO cells did not accumulate appreciable amounts of either protein upon proteasome inhibition, indicating that PEG10-RF1/2 and TRIM32 are indeed degraded through a Ubqln-dependent pathway. Chloroquine treatment had a negligible effect on the accumulation of these proteins (Fig. 3*B*).

Despite the accumulation of ubiquitin-conjugated CXX1B/RTL8 upon proteasome inhibition of WT HCT116 cells (111), its levels decreased in *Ubqln2*-deficient cells (Fig. 3*A*), in accord with the mouse data (Fig. 2). UBQLNs have occasionally been reported to protect some clients from protein degradation, although evidence for this has largely been limited to cell culture systems involving UBQLN overexpression (112). Inhibition of proteasomal degradation resulted in only a minor accumulation of RTL8 protein in TKO cells (Figs. 3*B* and S10*B*). Therefore, WT and TKO HEK 293 cells were incubated with high-dose proteasome inhibitors as in Figure 3*B*, which partially rescued TKO RTL8 levels, indicating that its abundance is at least in part regulated by proteasomal degradation (Fig. S10*B*).

Since proteasome inhibition did not entirely rescue RTL8 protein levels, we considered whether its synthesis was altered in the absence of UBQLNs. To examine RTL8 synthesis, WT or TKO HEK 293 cells were starved of methionine and incubated with the methionine analog azido-homo-alanine (AHA) to label the nascent protein (Fig. S11*A*). Following incubation, RTL8 protein was immunoprecipitated. Although the total RTL8 protein content in TKO cells is dramatically lower than in WT cells (Fig. S11, *A*–*B*), the amount of nascent RTL8 was indistinguishable at 30 min of labeling between the two genotypes (Fig. S11*B*), indicating that synthesis of RTL8 is not altered by the loss of Ubqlns.

To confirm the UBQLN dependence of the degradation of TRIM32, PEG10, and RTL8, we designed a set of fusion proteins with the photo-convertible fluorophore Dendra2 (113) at the C terminus with an IRES-cyan fluorescent protein (CFP) cassette as a control for transfection efficiency (Fig. S12*A*). These constructs were expressed in either WT or TKO HEK 293 cells. After 48 h of expression, the cellular pool of Dendra2-fusion proteins was photoconverted from green fluorescence to red fluorescent protein by exposure to 488 nm light (Fig. S12*B*). Flow cytometric analysis allowed the quantitation of CFP, green fluorescence, and photoconverted red fluorescence of cells at multiple timepoints following the laser pulse.

PEG10 protein degradation was dramatically delayed in the absence of UBQLN expression (Fig. 3*C*), with very little PEG10 protein degraded in TKO cells during the 24-h testing period. Results confirming PEG10's status as a direct client of UBQLN proteins were obtained by western blotting through cycloheximide chase assays performed on iNeurons expressing endogenous PEG10 protein (Fig. S12*C*). Very little RTL8C protein was degraded during the time course in transfected cells, although TKO cells had a modest acceleration in early RTL8C protein loss (Fig. 3*D*), consistent with the hypothesis that UBQLN2 protects CXX1B/RTL8 from degradation. As with PEG10, TRIM32 degradation was also delayed in TKO cells (Fig. 3*E*). Taken together, these results strongly suggest that PEG10 and TRIM32 are *bona fide* UBQLN2 clients, whereas CXX1B/RTL8C is protected from degradation by UBQLN2.

### Pathway analysis of *Ubqln2*^*−/−*^ tissue reveals *in vivo* neuronal defects in serotonergic signaling

**Figure 4:**
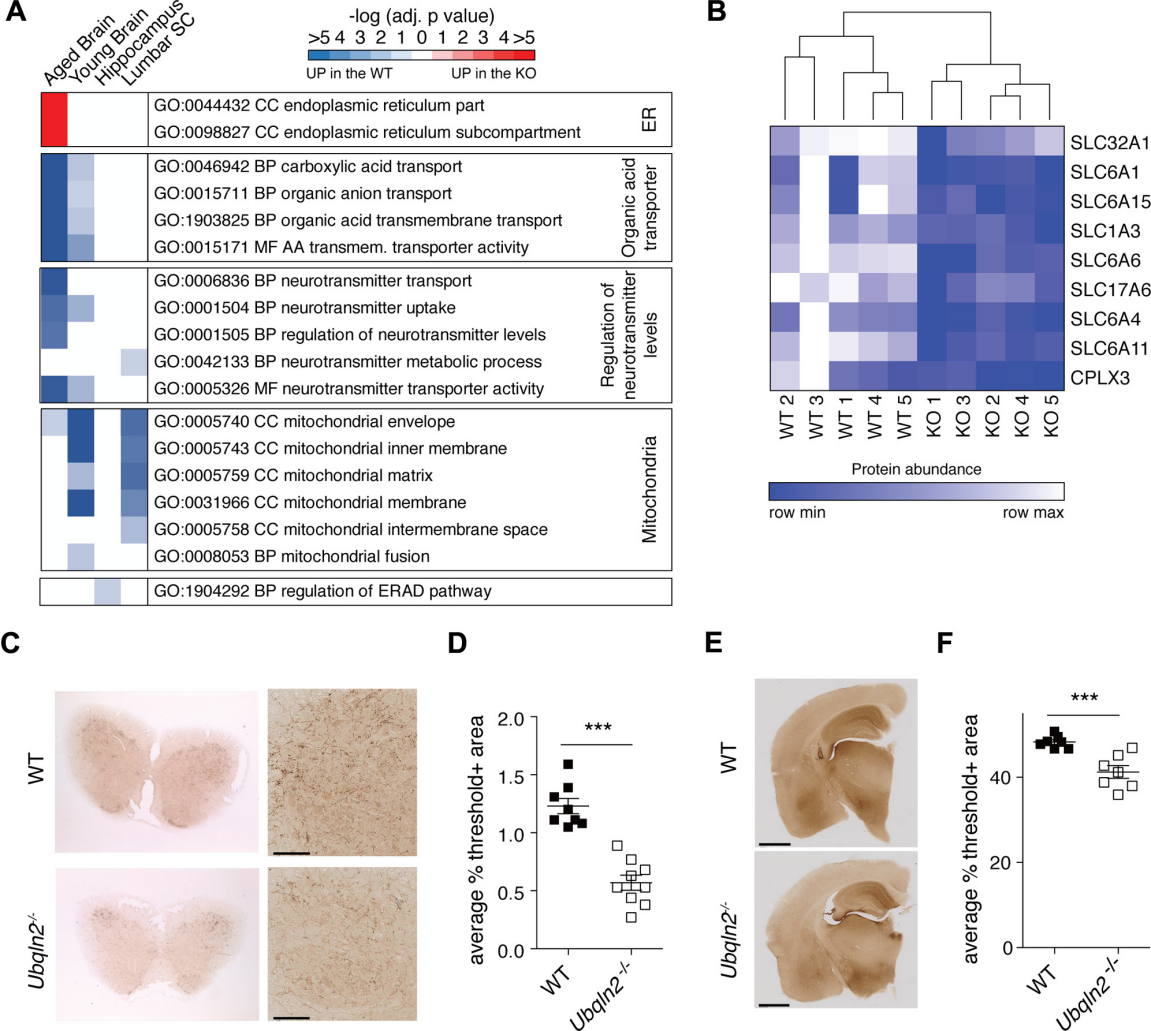
**Serotonin staining is decreased in aged *Ubqln2***^***−/−***^**tissues**. *A*, GO-term enrichment analysis was performed on brains, hippocampus, and lumbar spinal cords from aged and young mice (from Fig. 2) to identify pathways that were altered upon *Ubqln2* loss. Color corresponds to the significance of pathway enrichment as measured by the -log_10_(adjusted *p*-value) and is either *red*, for pathways enriched upon *Ubqln2* loss (Up in KO), or *blue*, for pathways downregulated upon *Ubqln2* loss (Up in WT). Notable significantly enriched pathways were selected from Table S5 for graphic representation. *B*, GO-term enrichment of brain hemispheres from aged WT and *Ubqln2*^*−/−*^ animals (shown as KO) reveals a decrease in expression of proteins involved in neurotransmitter signaling. Nine significantly altered proteins from the Molecular Function (MF) GO-term:0005326 neurotransmitter transporter activity were highlighted to demonstrate differences between WT and *Ubqln2*^*−/−*^ counterparts. Color represents protein abundance as measured by mass spectrometry, scaled independently for each protein. *C*, lumbar spinal cords from approximately 1-year-old animals were prepared for histological quantitation of serotonin-positive neuronal projections *via* serotonin staining. Shown are representative images of spinal cord cross section (*left*) and zoomed in area (*right*) highlighting neuronal projections. The scale bar represents 100 μm. *D*, quantification of serotonin positivity as a proportion of area from (*C*) using thresholding. n=8 to 9 animals. *E*, coronal sections of brains from young animals were prepared for histological quantitation of serotonin-positive neuronal projections. The scale bar represents 1 mm. *F*, quantification of serotonin positivity as a proportion of area using thresholding to determine serotonin positivity. n = 7 animals. Statistical significance for (*D* and *F*) was determined using an unpaired, two-tailed Student's *t* test. BP, Biological Process; CC, Cellular Component.

Global proteomic analysis can be used to identify pathways that link a genetic perturbation to the development of overt phenotypes *in vivo*. We performed GO-term enrichment on all proteins that were either upregulated or downregulated significantly in *Ubqln2*^*−/−*^ animals. Several pathways were implicated in this analysis (Table S5), the most notable of which are highlighted in Figure 4*A*. Upregulation of proteins involved in endoplasmic reticulum stress (see also [114]) was evident in aged *Ubqln2*^*−/−*^ brains (Fig. 4*A*) but not in young tissues (Table S5). One cluster of closely related pathways involving neurotransmitter proteins and their transporters was downregulated in both aged and young *Ubqln2*^*−/−*^ brains (Fig. 4*A*). To examine this more closely, proteins assigned to the GO-term neurotransmitter transporter activity were quantified and compared in aged brains of WT and *Ubqln2*^*−/−*^ littermates for their relative expression (Fig. 4*B*). Among these proteins, the serotonin transporter SLC6A4 (also known as SERT or 5-HT) was one of the most affected (Fig. 4*B*). To confirm alteration of this pathway, spinal cords and brains of WT and *Ubqln2*^*−/−*^ aged animals were immunostained for serotonin (Fig. 4, *C* and *E*), which was found at significantly lower levels in *Ubqln2*^*−/−*^ tissues as compared with the WT counterparts (Fig. 4, *D* and *F*).

### Proteomics of ALS disease models with *Ubqln2* mutation

**Figure 5:**
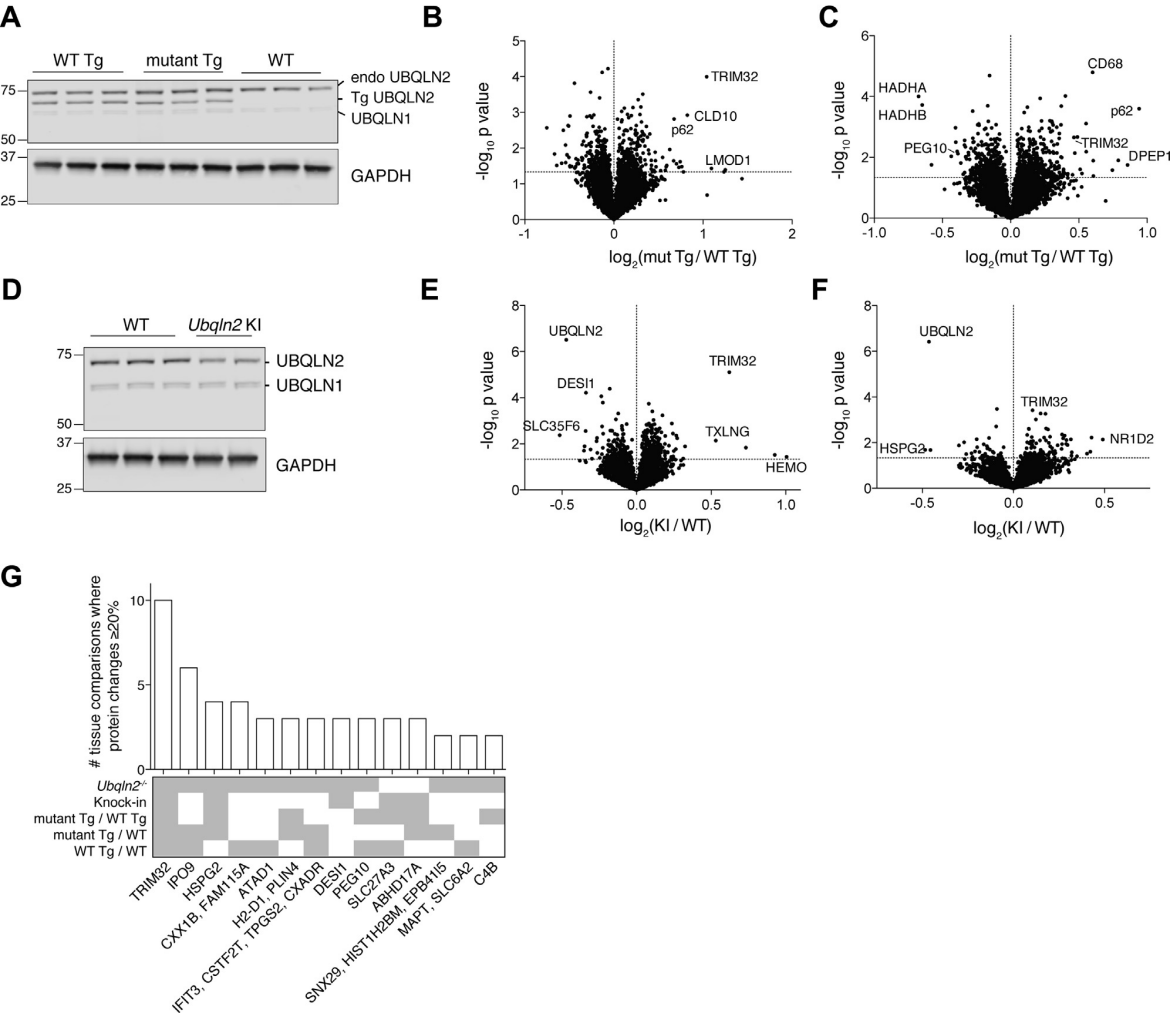
**Proteomic meta-analysis of multiple *Ubqln2*-mediated mouse models of ALS**. *A*, western blot of endogenous UBQLN2 (“endo,” *top band*), human UBQLN2 transgene (“Tg,” *middle band*), and UBQLN1 protein (*bottom band*) in hippocampal tissue of WT transgenic (WT Tg), mutant (P497S) Tg, and nontransgenic (WT) mice. GAPDH is a loading control. *B* and *C*, Hippocampus (*B*) and lumbar spinal cord (*C*) from the same animals used in (*A*) were processed for tandem mass tag analysis as in Figure 2. n = 3 animals per genotype. *D*, western blot of UBQLN2 and UBQLN1 in hippocampus of WT and *Ubqln2* knock-in (KI) (P520T) mice. Hippocampus (*E*) and spinal cord (*F*) of WT or mutant *Ubqln2* KI mice. n = 3 WT animals, 2 KI animals. *G*, individual proteins upregulated or downregulated at least 20%, allowing for standard error, in multiple tissues and/or model systems. The *y*-axis shows the number of tissues in which the protein is altered, and the *x*-axis those proteins' names. Below the bar graph is a graphical summary of the genetic backgrounds in which the protein is altered.

ALS-linked point mutations of *UBQLN2* could in principle be nullimorphic, hypomorphic, hypermorphic, or neomorphic. To assess this at the whole-proteome level, we analyzed isolated hippocampus and spinal cord from transgenic Ubqln2 animals that overexpress the WT or P497S allele of human *UBQLN2* (59). Tissues were taken at a young age (Table S1) to minimize downstream effects of late-stage disease and to facilitate the identification of client proteins before they are obscured by stress responses associated with disease progression. The comparison by TMT of these two models with nontransgenic animals showed a distinct clustering of biological triplicates in both hippocampal and spinal cord samples (Fig. S13, *A*–*B*). We quantified the amount of total UBQLN2 in WT *versus* transgenic animals using peptides that are identical between mouse and human UBQLN2. The hippocampal tissue of WT Tg mice had approximately twice as much UBQLN2 protein as control animals, and the degree of overexpression was statistically indistinguishable between animals expressing mutant and WT transgenes (Fig. S13*C*), as additionally validated by western blotting (Fig. 5*A*).

As a control for effects of overexpression, we initially compared hippocampus and spinal cord proteomes of transgenic mice overexpressing WT human UBQLN2 with those of nontransgenic animals (Fig. S13, *D*–*E*). Numerous hits were found, which was unexpected, given the modest degree of overexpression. Thus, UBQLN2 function appears to be highly sensitive to its levels. The transgene reduced TRIM32 levels in both hippocampal and spinal cord samples, further supporting the view that TRIM32 is a major UBQLN2 client. CXX1B levels were elevated upon overexpression of *Ubqln2*, consistent with the hypothesis that UBQLN2 protects CXX1B from degradation, as suggested above. Results from the transgenic animals (Fig. 5, *A*–*C*) showed an elevation of TRIM32 as a result of the P497S mutation, suggesting that this mutation compromises shuttling of TRIM32 to the proteasome for degradation. It is surprising that this was not the case for PEG10, which was reduced in level by the mutation. Thus, the mutational effects are client protein specific in the context of this transgenic model. Overexpression of the mutant allele of *UBQLN2* also caused enrichment of SQSTM1/p62 in both the hippocampus and spinal cord (Fig. 5, *B* and *C*), which was confirmed by western blotting (Fig. S13, *F*–*G*).

We proceeded to examine another characterized mouse model (33), which is based on KI rather than a transgenic overexpression of a mutant allele. The proteomic impact of the P520T KI mutant was weaker than that of the transgenic mutant or the null, perhaps due in part to a partial reduction in the UBQLN2 level caused by the mutation (Fig. 5, *D*–*F*). Other than TRIM32, the proteomic hits between transgenic and KI models were largely distinct (Fig. 5).

### Meta-analysis of proteomic datasets

Individual proteins were assessed for altered levels in all tested *Ubqln2*-mediated models of neurodegenerative disease. Proteins altered at least 20% in at least two tissues examined are highlighted in Figure 5*G*. TRIM32 was the most widely shared protein, with at least 20% alteration in all tissues tested, with representation in each genetic model examined. The nuclear importer IPO9 was shared by six tissues among *Ubqln2*^*−/−*^ animals, as well as *Ubqln2*-transgenic model systems. The retroelement proteins CXX1B and PEG10 were altered in *Ubqln2* transgenic mouse tissues in addition to the *Ubqln2*^*−/−*^ tissue (Figs. 5*G* and S13, *C*–*D*). Thus, interestingly, some features of the null mutant were observed upon overexpression of the WT protein.

**Figure 6:**
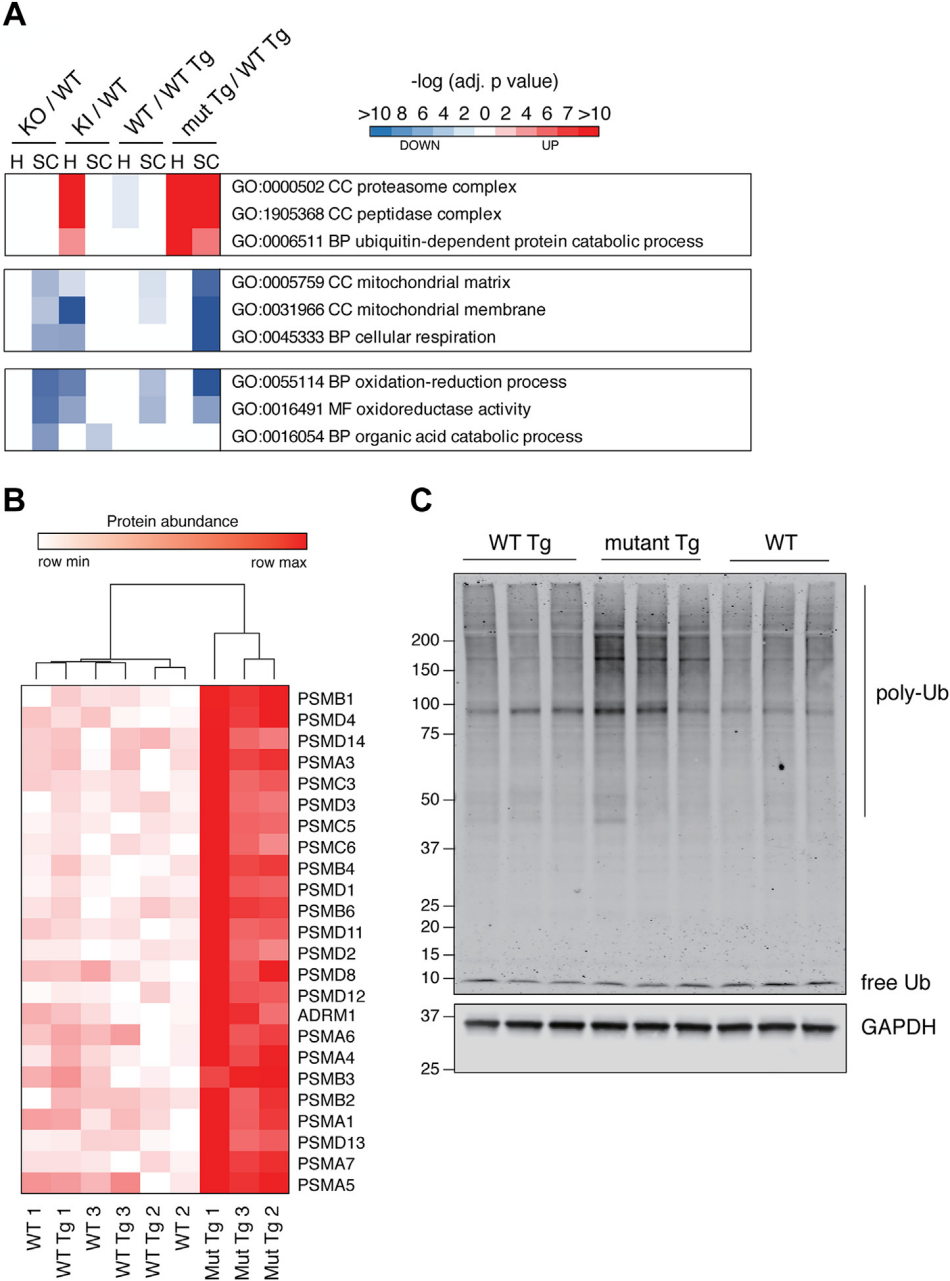
**Upregulation of the ubiquitin-proteasome system in *Ubqln2*-Tg animals**. *A*, GO-term enrichment analysis was performed on hippocampus (H) and lumbar spinal cord (SC) from *Ubqln2*^*−/−*^, *Ubqln2* KI (P520T), *Ubqln2* WT transgenic, or *Ubqln2* (P497S) mutant transgenic mice compared with their respective controls to identify pathways that were altered upon *Ubqln2* perturbation, as in Figure 4. The most significantly enriched pathways were selected for visual summarization from Table S4. *B*, GO-term GO:0000502 Cellular Compartment (CC) proteasome complex breakdown of individual proteins in hippocampus from Tg animals (similar to Fig. 4*B*). *C*, western blot against ubiquitin (Ub) shows accumulation of ubiquitin conjugates (poly-Ub) in mutant *Ubqln2*-transgene (mutant Tg) expressing hippocampal tissues of mice. GAPDH is a loading control. BP, biological process; MF, molecular function.

In the second form of meta-analysis, a similar GO-term enrichment to that in Figure 4 was performed on the hippocampal and spinal cord tissue using proteins significantly altered between WT and mutant conditions (Fig. 6*A*). Some metabolic pathways were downregulated in *Ubqln2* mutants, reflecting subtle changes to proteins involved in the generation of molecules involved in serotonergic and other neurotransmitter signaling (Table S5). However, there was also downregulation of pathways enriched for mitochondrial proteins, especially upon overexpression of the mutant UBQLN2 protein (Fig. 6*A*). Finally, mutant UBQLN2 protein expression caused an upregulation of proteasomal degradation pathways, including many proteasome components, as well as autophagy-directed factors such as SQSTM1/p62 (Table S5, Figs. 5, *E*–*G* and 6*A*). A closer investigation of the cellular compartment GO-term pathway “Proteasome complex” revealed an upregulation of many proteasome subunits and accessory proteins in the hippocampus of transgenic mice expressing mutant *UBQLN2* (Fig. 6*B*). Western blotting of hippocampal tissue from transgenic mice confirmed the accumulation of ubiquitin conjugates (Fig. 6*C*), consistent with (59), and suggests that upregulated proteasome components may be a response to a broad defect in proteasome function as suggested by the accumulation of ubiquitinated proteins upon UBQLN2 dysfunction.

## Discussion

As Ubqln proteins are substrate delivery factors for the proteasome, client protein stabilization has been suggested to underlie the development of disease (47). However, the physiological clients of UBQLN2 in neuronal cells remained poorly understood and had not been studied in an unbiased, global manner. We performed the first large-scale global proteomic analysis of *Ubqln2*-mediated neurodegenerative disease, employing multiple models to identify proteins that are under the control of UBQLN2. Quantitative global proteomics offers a view of the mutant or disease phenotype that is exceptional in detail and in scope. Moreover, because we have characterized the *Ubqln2*^*−/−*^ mutant as a baseline, our proteomic data provide strong evidence that the etiological mutations studied here result in both loss-of-function and gain-of-function effects, which interestingly depend on the target protein. For example, since the TRIM32 levels were elevated in *Ubqln2* null mutants, reduced in *Ubqln2* overexpressors, and elevated by *Ubqln2* point mutations, stabilization of TRIM32 is not a purely neomorphic phenotype. But, although PEG10 is, like TRIM32, elevated in the null, it is, unlike TRIM32, decreased by the P497S mutation. Most likely the P497S mutation attenuates delivery of TRIM32 to the proteasome but does not do so for PEG10.

Although previous studies have suggested that UBQLNs can regulate the turnover of a significant fraction of proteasome substrates (30, 31, 34), the changes to the proteome that we find in disease-relevant tissue *in vivo* are instead focused on a limited number of proteins. Although our proteomics approach is global in scope, it may still miss major clients if an explicit stress needs to be imposed to elicit their degradation, or if the mixed cell populations taken from tissues dampen effects that are restricted to specific cell types. Among the strongest hits in our experiments were TRIM32, PEG10, and CXX1B, none of which had previously been identified as UBQLN2 clients. Perturbations of these proteins were found to be mediated by changes in their rates of degradation, at least in part through the proteasome, although this does not exclude the participation of other proteolytic pathways, particularly for CXX1B. To our surprise, however, the strongest hit globally, CXX1B, is actually reduced in level upon loss of UBQLN2 function, indicating that UBQLN2 inhibits rather than promotes CXX1B degradation. The Ubqln effect on CXX1B is very robust as it was observed in murine brain and spinal cord, as well as in human HEK 293 cells and iNeurons (known as RTL8 in humans). Although the mechanism of CXX1B destabilization remains under investigation, the findings indicate that, depending on the target protein, UBQLN2 is capable of both positive and negative regulation.

A common observation among neurodegenerative conditions is that protein aggregates accumulate in cells before the onset of overt disease. By comparing aged and young animals of the same genetic background, we were able to generate a snapshot of both early and late proteomic changes to relevant disease-related tissues. TRIM32, which was a strong hit in multiple *Ubqln2*-dependent models of disease, was altered even in young brain tissue, as was CXX1B. However, pathway analysis of young brains showed much less involvement of metabolic proteins compared with their aged counterparts. Based on these findings, the increase in TRIM32 and concomitant decrease in CXX1B may be sensitive early indicators of developing *Ubqln2*-dependent neurodegenerative disease. Further work is underway to see how broadly these changes are found in unrelated cases of sporadic ALS and other neurodegenerative conditions.

In some tissues tested, the most highly upregulated protein upon *Ubqln2* alteration was PEG10; conversely, CXX1B was often the most downregulated protein. Both proteins are members of the Mar retroelement family, which also includes eight other related proteins. Mar retroelements are missing key features of active retrotransposons: they lack the necessary elements for transposition in the genome, and its family members are not arranged in tandem but are instead dispersed over multiple chromosomes (70). Despite having lost ancestral functions, multiple members of the Mar family have undergone “neofunctionalization.” For example, LDOC1 regulates NFκB activity and sensitizes cells to apoptosis (115, 116). PEG10 is indispensable for placental development (75, 77) and regulates proliferation (76). Although the function of CXX1B/RTL8 remains unknown, the multiple genetic duplications that generated RTL8A-C implicate functional diversification (70). Given that UBQLN2 binds to both PEG10 and CXX1B/RTL8, and that PEG10 and CXX1B/RTL8 are similarly binding partners, we speculate that there may be regulatory interactions between the two retroelement proteins that govern their divergent fates upon *Ubqln2* perturbation.

TRIM32 regulates muscle atrophy through regulated degradation of the proteins dysbindin (64) and actin (65). In addition, TRIM32 expression induces neuronal differentiation from precursor cells (61). Mutations in the RING domain of TRIM32 cause muscular dystrophy (63), but mutations in the BBS domain of the protein cause the developmental disorder Bardet-Biedl syndrome (62), indicating that the protein has diverse roles in CNS development and function. TRIM32 expression is elevated at the mRNA level upon a variety of CNS insults, including Alzheimer's disease (117), Duchenne's muscular dystrophy (118), and acute spinal injury (119). In this study, we find a different, posttranslational form of TRIM32 regulation mediated by UBQLN2.

By performing pathway analysis on our proteomic results, we were able to identify groups of proteins whose abundance changed upon *Ubqln2* perturbation. One example of this is the downregulation of proteins involved in setting neurotransmitter levels in both young and aged *Ubqln2*^*−/−*^ brains. Validation of this pathway's downregulation was performed through histological analysis of brains, which indeed showed decreased serotonin staining in *Ubqln2*^*−/−*^ tissues. These findings provide an intriguing link between dysregulation at the protein level and the emergence of neurologic phenotypes. Decreased serotonin has previously been observed in the plasma, cerebrospinal fluid, and spinal cords of patients with ALS, and loss of serotonergic neurons leads to spasticity in mouse models of disease, as measured by hind limb clasping (120).

A second pathway that emerged upon analysis was the upregulation of protein degradation pathways in animals expressing the mutant but not wildtype transgene (Fig. 6*A*). Dozens of proteasome components were upregulated (Fig. 6*B*), most likely as a compensatory response to deficient output of the ubiquitin-proteasome pathway. Indeed, these animals have ubiquitin-positive inclusions in neurons of the ventral horn and increased poly-ubiquitin conjugates in homogenized CNS tissue (59), which our results confirm (Fig. 6*C*). The selective autophagy receptor p62 was also elevated in response to the mutant transgene (Fig. 5), consistent with a perturbation of selective autophagy and thus a general dysfunction of protein degradation. Finally, as UBQLN2 could potentially have client proteins that it does not target for degradation—interactions that would not be easily detected by global proteomics—we performed proximity labeling of UBQLN2 by the APEX2 method. The results implicated UBQLN2 but not its paralog UBQLN1 in endosomal trafficking and signaling functions that may be proteasome independent (Fig. S9 and Supplemental Discussion).

In conclusion, this study has identified novel clients of UBQLN2 and provided a new understanding of the proteomic landscape of *Ubqln2*-mediated ALS. By leveraging the technology of global proteomics, we have identified proteins that change upon *Ubqln2* perturbation in disease-relevant tissues. Notably, these hits were shared among multiple models of *Ubqln2*-mediated disease, strongly linking them to *Ubqln2* despite fundamental differences in the nature of the genetic perturbation. Furthermore, multiple hits were also altered in human models of *UBQLN2* loss. Future studies of *Ubqln2*-mediated ALS will apply quantitative global proteomics to patient-derived samples, and explore the potential effects of TRIM32, PEG10, and RTL8 on neuronal function.

## Experimental procedures

### Antibodies and reagents

Information on antibodies used in this study can be found in Table S6.

### Cell lines

HEK 293 cells lacking *UBQLN1*, *2*, and *4* (TKO) were provided by Dr Ramanujan Hegde (Medical Research Council Laboratory of Molecular Biology; [30]). They were maintained in Dulbecco's modified Eagle's medium (Corning) with 100 U/ml penicillin/streptomycin (Gibco), 12.5 mM L-glutamine (Gibco), and 10% heat-inactivated FBS (Hyclone).

Human ES cells (H9, WiCell Institute) were cultured in fresh E8 medium (121) on tissue culture plates coated with Matrigel; the medium was changed daily and cells were passaged at roughly 50% confluency approximately every 3 days with 0.5 mM EDTA in sterile PBS. The SpCas9 protein generated from the expression plasmid pET-NLS-Cas9-6xHis (Addgene #62934) was purified according to (122). Single guide RNA was generated using the GeneArt Precision gRNA Synthesis Kit (Thermo Fisher) according to the manufacturer's instructions and was purified using an RNeasy Mini Kit (Qiagen). To generate *UBQLN2*-deficient ES cells, 0.6 μg of the single guide RNA targeting sequence GCCTAAAATCATCAAAGTCA was incubated with 3 μg SpCas9 protein for 10 min at room temperature and electroporated into 2 × 10^5^ H9 cells. Mutants were identified by Illumina MiSeq and further confirmed by western blotting. Following confirmation of genetic deletion of *UBQLN2*, WT and *UBQLN2*^*−/−*^ ES cells were differentiated into neurons by infecting cells with Ngn2-expressing lentivirus as described (80). After 7 days of differentiation, cells were analyzed for expression of potential UBQLN2 clients.

### Animals

*Ubqln2* knockout animals in the C57BL/6 background were generated by insertion of *loxP* sites flanking the one exon of murine *Ubqln2*. Deletion of *Ubqln2* was induced by transient expression of Cre in ES cells, which were then used to generate live animals. Mice were genotyped using tail clips and the Qiagen DNA extraction kit with FastCycle PCR kit. *Ubqln2*^*−/−*^ mice were housed according to Institutional Animal Care and Use Committee guidelines and in compliance with the Institute for Lab Animals' guidelines for the humane care and use of laboratory animals. All experimental *Ubqln2*^*−/−*^ and WT littermate mice, as well as *Ubqln2* KI and WT littermate mice (33), were males to eliminate the confounding variable of sex owing to the presence of the *Ubqln2* gene on the X-chromosome (Table S1). For the sake of simplicity, *Ubqln2*^−/y^ animals are designated as *Ubqln2*^−/−^. *Ubqln2* Tg mice (59) were all heterozygous for the *Ubqln2* transgene. For brain and spinal cord tissue collection, mice were anesthetized with 2.5% tribromoethanol (0.5 ml/25 g body weight) and transcardially exsanguinated with phosphate-buffered saline (PBS). Dissected brain and brain hemispheres included intact olfactory lobe, cerebral cortex, and cerebellum. All other euthanasia was performed with CO_2_ according to Institutional Animal Care and Use Committee standards, followed by cervical dislocation.

### Behavioral testing

#### General

The order of animal testing was randomized by genotype to avoid potentially confounding effects of recording chambers, arenas, or time of day. Animals were also acclimated to the anteroom of the experimental room (active avoidance [AA] and fear conditioning [FC] experiments) or the experimental room (all other tests) prior to the beginning of each experiment, and all animals were tested during the day. Between tests, experimental chambers and arenas were cleaned using water or ethanol-containing wipes. To avoid any training sequence effects between AA and FC experiments, the order of AA and FC experiments was also counterbalanced: while half of the WT and *Ubqln2*^*−/−*^ mice first underwent FC testing followed by AA testing, the remaining half first underwent AA testing instead. Male mice used for behavioral testing were housed individually for the duration of all tests and experimenters were blinded to animal genotype for the duration of testing.

#### Open field

An automated Photobeam Activity System Open Field recording system (San Diego Instruments) was used to record spontaneous locomotor activity. Recordings were conducted in a room with bright lights. Mice were placed individually in transparent Plexiglas chambers (40.5 × 40.5 × 38 cm), and horizontal and vertical movements were recorded by two frames in the chamber that were fitted with infrared beams. Activity was measured over 60 min by calculating the total number of beam breaks and rearings per session beginning with the placement of mice into the Plexiglas chambers. Consecutive open field sessions were separated by 3 days.

#### Hind limb clasping

Hind limb clasping was performed by grasping the mouse's tail near its base and suspending the mouse in the air, clear of all surrounding objects for 20 s. Scoring was conducted as previously described (123).

#### Wire hang

Mice were placed on a wire grate and allowed to grasp the wires, after which the grate was gently inverted. The time the mouse was able to hang suspended was recorded, with a maximum latency of 60 s. Mice unable to reach the full 60 s could repeat up to 3 times, after which their maximal score was recorded.

#### Balance beam

Wooden dowels, 104 cm long (of large, medium, and small diameter), were elevated 50 cm above bench surface and angled upward toward an enclosed escape box. To begin training on balance beams, each mouse was first placed in the escape box for 30 s, then placed at the start end of the largest balance beam of 24 mm diameter. Mice had four consecutive training trials with a maximum acceptable time limit of 1 min to cross. Testing was conducted 2 days after training and consisted of nine trials: three on the large beam, three on the medium (19 mm diameter) and three on the small (11 mm diameter). The time required to cross an 80-cm-long distance to the escape box on the beam, as well as hind paw foot slips, was recorded for each trial.

#### Fear conditioning

FC was conducted according to (124). FC chambers for mice (30 × 24 × 24 cm) were used inside of sound-attenuating cubicles (Med Associates). Each chamber was equipped with a house light, an IR light, a white noise speaker, and a near-IR camera to record movement. The floors of the compartments were equipped with stainless steel metal grids connected to a shock generator. The experiment took two consecutive days: training on day one and a contextual test on the morning of day two followed by a cued test in the afternoon. Furthermore, recording settings of hardware and data acquisition software (Video-Freeze software, Med Associates) were readjusted to account for the different lighting and background conditions for the camera during the cued test. Video data were acquired at a rate of 30 frames per second, the observation interval was 15 frames or 0.5 s, and the observation duration was 3 frames or 0.1 s. The motion threshold was set to 20 artificial units (AU). Data were acquired by the discrete method: if the motion index was below 20 AU throughout a recording period, freezing was registered. Percent freezing was defined as the percentage of recording periods in which freezing occurred relative to the total number of observation periods. The system was calibrated using camera recordings from empty chambers before the mice were placed inside. FC training began with 3 min of baseline recording, followed by two trials of a 2-s shock (0.7 mA), which served as unconditioned stimuli (UCS) and 30 s of white noise (∼90 dB; background noise: ∼65 dB), which initially served as the neutral stimulus or conditioned stimulus. Neutral stimulus or conditioned stimulus coterminated with the UCS. The two trials were separated by 90 s (offset to onset), and the second trial was followed by another 90 s of recording to measure freezing at the end of the training period. During contextual FC, each animal was reintroduced into the same unaltered chamber where training occurred for a 5-min recording period during which neither white noise nor shock was given. Cued FC testing was conducted in an altered context: for the cue, the house lights were off, the gridded floor was covered with plastic, an insert was placed to alter chamber walls, and the scent of the chamber was changed with a small amount of 1% acetic acid (Mallinckrodt Chemicals). Testing consisted of 3 min of baseline recording, followed by 3 min of white noise without shock. The percent time spent freezing was calculated for each of these experimental phases.

For data analysis, the average motion index across the five 2-s bins prior to the shocks served as (pre-UCS) baseline relative to the response during the actual 2-s UCS (during-UCS). The number of fecal boli left in the chamber after the cued-FC test period was used as a proxy measure of anxiety and emotionality.

#### Active avoidance

AA experiments were performed according to (124) using two-way avoidance chambers inside of sound-attenuating cubicles (Med Associates). Each chamber had two compartments (21 × 16 × 25 cm) that were connected by an auto-guillotine door, and each compartment was equipped with stimulus light and tone generator, as well as four parallel IR light beams located 2.5 cm above the grid floor. Like chambers for FC experiments, the floors of the compartments were connected to a shock generator *via* stainless steel metal grids. The experimental task was performed according to (125), where each animal received two training sessions, consisting of 100 trials each, on two consecutive days.

Sessions began by placing a mouse into one of the two compartments (selection of which was randomized across the cohort of animals) and letting the mouse acclimatize for 5 min with the door between the compartments closed and all lights off inside the enclosure. Afterward, each trial began by opening the guillotine door, turning on the stimulus light, and activating the tone (approximately 73 dB). Background noise was approximately 68 dB. If the animal crossed into the second compartment within 5 s of stimulus, the door was closed, the light and the tone were switched off, an avoidance response was recorded, and the intertrial interval (ITI) began. If, however, the mouse did not cross within 5 s, a shock (0.3 mA, 2 s) was delivered. If the mouse crossed within 2 s, the shock was stopped, the door was closed, the light and the tone were switched off, an escape response was recorded, and the ITI began. If, however, the mouse did not cross within 2 s, the shock was stopped, the door was closed, the light and the tone were switched off, an escape failure was recorded, and the ITI began. The ITIs ranged from 25 to 55 s and averaged 40 s. After the ITI was completed, the next trial was started. This trial-ITI sequence was continued until 100 trials were completed. Hardware and data acquisition were controlled by Med-PC software (Med Associates). Percent avoidance, percent escapes, and percent escape failures were calculated using average values obtained from only 5 blocks of 20 consecutive trials, thereby enabling ANOVA at a limited number of factor levels for 100 individual trials.

### Sample preparation for mass spectrometry analysis

Tissues were lysed and homogenized in 8 M urea buffer (8 M urea, 150 mM NaCl, 50 mM Hepes [pH 7.5], 75 mM NaCl, 1x EDTA-free protease inhibitor cocktail [Roche], 1x PhosSTOP phosphatase inhibitor cocktail [Roche]). Lysates were centrifuged at 17,000*g* for 15 min at 4 °C and protein concentration quantified by bicinchoninic acid assay ([BCA] Pierce). A total of 100 μg of protein of each sample was collected and brought to 1 μg/μl in 8 M urea buffer. For reduction and alkylation of cysteines, protein extracts were sequentially incubated with 5 mM TCEP at RT for 30 min, 14 mM iodoacetamide at RT for 30 min in the dark, and 10 mM dithiothreitol for 15 min at RT. Finally, proteins were methanol–chloroform precipitated and the protein pellet was resuspended in 200 mM EPPS (pH 8.5). For digestion, LysC (Wako) was added at 1:100 (LysC:protein) ratio and incubated overnight at RT in an orbital shaker at 1500 rpm. The day after, trypsin (sequencing grade modified trypsin, Promega) was added at 1:75 (trypsin:protein) ratio and incubated for 5 h at 37 °C with shaking at 1500 rpm. After digestion, samples were clarified by centrifugation at 17,000*g* for 10 min and the peptide concentration was quantified using a quantitative colorimetric peptide assay (Thermo Fisher). For TMT labeling we used TMT10-plex kits (Thermo Fisher). In brief, 25 μg of peptides were brought to 1 μg/μl with 200 mM EPPS (pH 8.5), acetonitrile (ACN) was added to a final concentration of 30%, and then 50 μg of each TMT reagent was added (information on all TMT sample labels is included in Table S1), followed by incubation of the mixture for 1 h at RT. Finally, the reaction was stopped by the addition of 0.3% hydroxylamine (Sigma) for 15 min at RT.

To check mixing ratios and labeling efficiency, samples (1 μg each) were pooled, desalted, and analyzed by mass spectrometry in a Q-Exactive (Thermo Fisher). Normalization factors calculated from this “label check” (Table S3) were used to mix the rest of the samples before desalting with tC18 50 mg SepPak solid-phase extraction cartridges (Waters) and drying using a SpeedVac. Next, dried peptides were resuspended in 10 mM ammonium bicarbonate 5% ACN (pH 8.0) and fractionated with basic-pH reversed-phase high-performance liquid chromatography (using a 3.5-μm Zorbax 300 Extended-C18 column [Agilent]). Fractions were collected in a 96-well plate, then combined into 24 samples. Twelve of these were desalted following the C18 Stop and Go Extraction Tip (STAGE-Tip) and dried down in a SpeedVac. Finally, peptides were resuspended in 1% formic acid, 3% ACN, and analyzed by LC-MS3.

### Liquid chromatography-MS3

All samples were analyzed with an LC-MS3 data collection strategy (126) on Orbitrap Fusion or Orbitrap Fusion Lumos mass spectrometers (Thermo Fisher) equipped with a Proxeon Easy nLC 1000 for online sample handling and peptide separations. Samples resuspended in 1% formic acid, 3% ACN were loaded onto a 100-μm inner diameter fused-silica microcapillary with a needle tip pulled to an internal diameter less than 5 μm. The column was packed in-house to a length of 35 cm with a C_18_ reverse phase resin (Accucore 150 2.6 μm, 150 Å, Thermo Fisher). Peptides were separated using a 150- or 135-min linear gradient from 3% to 25% buffer B (90% ACN + 0.125% formic acid) equilibrated with buffer A (3% ACN + 0.125% formic acid) at a flow rate of 400 nl/min across the column. The scan sequence for the mass spectrometer began with an MS1 spectrum, followed with the “Top N” (the top 10 precursors) isolation in the quadrupole, CID fragmentation, and MS2 detection in the ion trap. Finally, the top ten fragment ion precursors from each MS2 scan were selected for MS3 analysis (synchronous precursor selection, SPS), in which precursors were fragmented by HCD prior to Orbitrap analysis. More information related to the mass spectrometer set up for each TMT is included in Table S3.

### TMT-SPS-MS3 data analysis

Raw data were converted to mzXML format using a modified version of RawFileReader (5.0.7). A suite of in-house software was used to perform searches with SEQUEST (v28 revision 12) against a mouse or human target-decoy database downloaded from UniProt in July 2014 and February 2014, respectively. Mouse and human databases contained 51,547 and 88,479 entries, respectively. The most common contaminants were also included in the database. Database search criteria were as follows: tryptic with two missed cleavages and two variable modifications per peptide, a precursor mass tolerance of 20 ppm, fragment ion mass tolerance of 1.0 Da, static modifications of cysteines (alkylation, 57.0215 Da), TMT labeling of lysines and N-termini of peptides (+229.1629 Da), and variable oxidation of methionine (15.9949 Da). Peptide-spectrum matches were adjusted to a 1% false discovery rate using a linear discriminant analysis (127). Proteins were further collapsed to a final protein-level false discovery rate of 1% and assembled by principles of parsimony to produce the smallest set of proteins necessary to account for all observed peptides (127, 128). List of all peptides identified in each TMT are included in Tables S7–S10. Each analysis used an SPS-MS3-based TMT method, which has been shown to reduce ion interference compared with MS2 quantification (129). TMT reporter ion intensities were measured using a 0.003-Da window around the theoretical m/z for each reporter ion in the MS3 scan. Peptide spectral matches with poor quality MS3 spectra were excluded from quantitation (<100 summed signal-to-noise across 10 channels and <0.7 precursor isolation specificity). Peptides with a CV < 20 % within each replicate group were used for protein quantification. Finally, quantifications were normalized assuming equal peptide loading for all samples in each TMT. UBQLN1 and UBQLN2 proteins were quantified using unique peptides. The final protein quantification results were used for pathway analysis. Enrichment of GO-terms (CC, Cellular Component; MF, Molecular Function; and BP, Biological Process) was performed using the clusterProfiler R package (130). For these analyses, all proteins significantly up- or downregulated between WT and littermate mutant animals were selected. For the GO analysis of each TMT, all proteins quantified in the TMT were used as background.

### Histology and staining

Both the lumbar spinal cord and the left brain hemisphere, including intact olfactory lobe, cerebral cortex, and cerebellum, were drop-fixed in 4% paraformaldehyde for 2 days at 4 °C with agitation and then transferred to PBS for histopathological analyses. Lumbar spinal cord segments and hemi-brains were cryopreserved and were multiply embedded into gelatin matrixes using MultiBrain Technology (NeuroScience Associates). The lumbar spinal cord block was sectioned coronally at 25 μm and the hemi-brain block was sectioned coronally at 35 μm, and sections were sampled according to (131, 132). Antibody staining was performed at concentrations indicated in Table S6. Nissl staining (Fisher Scientific T409-25) was performed according to manufacturer's recommendations.

### Imaging and quantification of stained sections

Brain and spinal cord tissue samples processed by NeuroScience Associates were imaged on a Leica SCN400 whole slide scanning system (Leica Microsystems) at 200x magnification. Matlab (Mathworks) running on a high-performance computing cluster was used for all whole slide image analysis performed in a blinded manner. Motor neuron counts and quantitation of CD68, GFAP, and Iba1 were performed as previously described (131). Analysis of serotonin staining was performed using color thresholds and morphological operations in Matlab. The percent CD68, GFAP, IBA1, and serotonin positivity for the entire section was calculated by normalizing the positive pixel area to tissue section area and averaged from 8 to 12 sections per animal. All image analyses were performed blind to genotype groups and all images, segmentation overlays, and data were reviewed by a pathologist.

### Metabolic labeling and immunoprecipitation

Confluent cells in 10-cm dishes were starved in methionine free medium (Gibco) for 45 min, then the medium was replaced with 2 μM AHA (Life Technologies) in methionine-free medium for either 30 min or 4 h. After incubation, cells were harvested with trypsin, washed, and lysed at a concentration of 2 × 10^7^/ml of lysis buffer containing 50 mM Tris-HCl (pH 7.4), 150 mM NaCl, and 1% Triton X-100, but no EDTA or β-mercaptoethanol (BME). After 30 min on ice, the lysate was cleared *via* centrifugation at 17,000*g* for 10 min at 4 °C. A volume of 50 μl of the cleared lysate was saved for total AHA quantitation and 500 μl of the cleared lysate was used for immunoprecipitation of RTL8.

To immunoprecipitate RTL8, 500 μl of the cleared lysate plus 500 μl fresh lysis buffer with protease inhibitors was incubated with 7 μg FAM127B antibody (Proteintech) overnight with rocking at 4 °C. Protein A agarose beads (Thermo Fisher) were equilibrated with TBS buffer for 2 h at 4 °C with rocking. After one wash, 20 μl of beads was added to each immunoprecipitation tube and rocked for 2 h at 4 °C to bind. Following incubation, tubes were spun down and the lysate was removed to test for clearance of RTL8. Beads were washed 3x with TBS buffer. Proteins with AHA incorporation (nascent proteins) were then labeled with PEG4 biotin-alkyne using 0.5 volumes of buffer, using the standard protocol of the Thermo Fisher Click-It protein labeling kit. After labeling but before protein precipitation, tubes were washed 3x with TBS buffer and RTL8 was eluted with 50 μl of 5x Laemmli buffer with BME, then heated for 7 min at 95 °C. Total protein from 50 μl of lysate was labeled with PEG4 biotin-alkyne according to manufacturer's instructions and resuspended in 8 M urea buffer.

A total of 3 μg of biotin-labeled protein, from the original lysate, as ascertained by BCA (Pierce) of resuspended urea samples, was run on a 4% to 20% Bis-tris gel with MES buffer (Thermo Fisher). A volume of 30 μl of RTL8 immunoprecipitate was run on a 4% to 20% Bis-tris gel with MES buffer. Western blots were visualized with Streptavidin IRdye 800 and anti-rabbit IRdye 680 (LICOR).

### Western blot

Tissue samples and HEK 293 cells were lysed in 8 M urea buffer. Lysates were centrifuged at 17,000*g* for 15 min at 4 °C and the protein concentration was quantified by BCA (Pierce). Laemmli sample buffer, 1x (supplemented with BME), was added to each sample prior to SDS-PAGE. iNeuron cell samples were lysed in 8 M urea buffer, and protein was methanol–chloroform precipitated. The protein pellet was resuspended in 2x Laemmli buffer supplemented with BME and heated for 7 min at 95 °C. Samples were run on a 4% to 20% Tris-glycine gel or 4% to 12% NuPage Bis-Tris gel (Thermo Fisher) and wet-transferred onto PVDF or nitrocellulose membrane at 100 V for 90 min in an ice bath. Membranes were blocked with TBS:Aquablock (East Coast Bio) at 1:1 ratio for at least 20 min and incubated with primary antibody overnight. After three 5-min washes, membranes were incubated for at least 30 min with secondary LICOR antibodies for imaging. After washing again 3x for 5 min each, reactive bands were visualized and quantitated using LICOR Odyssey imager and software. Antibody concentrations are indicated in Table S6.

### qPCR

For qPCR of brain and spinal cord tissue, frozen samples were homogenized and RNA was extracted using the Qiagen Lipid RNEasy kit with on-column DNA digestion. After RNA extraction, cDNA was generated using the ABI Biosciences One-Step cDNA synthesis kit. qPCR was performed with the following Life Technologies TaqMan reagents and Perfecta II mastermix. A total of 10 ng of cDNA was used as input for gene expression assays, which were run in triplicate with internal *Gapdh* controls using standard Fast cycling parameters of an ABI 7500 Fast real-time PCR system. Raw triplicate Ct values were averaged and normalized against *Gapdh*. TaqMan primer/probe sets for mouse tissues were as follows: *Gapdh* (VIC): Mm99999915_g1, *Trim32* (MGB): Mm00551733_m1, *Peg10* (MGB): Mm01167724_m1, *Cxx1b* (MGB): Mm03032892_gH. Primer/probe sets for human iNeurons were as follows: *GAPDH* (VIC):, *TRIM32* (MGB): Hs01045822_m1, *PEG10* (MGB): Hs01122880_m1, *RTL8A* (MGB, also known as FAM127B): Hs00602281_s1, *RTL8B* (MGB, also known as FAM127C): Hs03004887_s1, and *RTL8C* (MGB, also known as FAM127A): Hs05048780_s1.

### Construct design

The human version of the *TRIM32* gene was purchased from Harvard Medical School PlasmID repository. Human full-length *PEG10* encoding both reading frame 1 and reading frame 2, and human *RTL8C*, were cloned from isolated iNeuron cDNA. All three genes were then cloned into LifeAct-Dendra2 from Addgene (plasmid ID: 54694) replacing LifeAct as an N-terminal fusion with Gibson cloning (SGI). Then, an IRES-CFP cassette from pMSCV-IRES-CFP II (pMIC II, Addgene plasmid ID: 52109) was inserted 3ʹ to the Dendra2 fusion protein, again by Gibson cloning. DH5α chemically competent *Escherichia coli* were transformed with expression constructs according to standard protocols. Plasmids were purified using a Zymo midiprep kit and brought to 1 μg/μl in sterile, endotoxin-free water. Dendra2 and CFP expression was confirmed by flow cytometry of transfected cells.

The APEX2-*UBQLN1* construct was generated using the *UBQLN1* gene from (36). The APEX2-*UBQLN2* construct was generated using cDNA from WT *UBQLN2-*expressing HEK 293 cells (30). Both gene cassettes were inserted into a pCDNA3.1 plasmid containing a V5-APEX2-GS linker cassette using Gibson cloning.

### APEX2 labeling and purification

*UBQLN1*, *2*, and *4* TKO HEK 293 cells were transfected with UBQLN1 or UBQLN2 APEX2 constructs using polyethylenimine (PEI) at a 3:1 PEI:DNA ratio (Polysciences, Inc). APEX labeling and subsequent streptavidin pulldowns were performed as described previously (133). Briefly, cells were preincubated in media with 500 μM biotin–phenol (ApexBio) for 1 h and then labeled for 1 min by adding hydrogen peroxide to a final concentration of 1 mM. After 1 min, the labeling medium was aspirated and cells were washed 3x with a quenching buffer containing 5 mM Trolox, 10 mM sodium azide, and 10 mM sodium ascorbate in PBS. Cells were harvested and spun down at 200*g*, after which the cell pellets were snap-frozen. The cell pellets were lysed in 2 M sodium hydroxide with 7.5% BME (Sigma), and proteins were then pelleted after TCA precipitation. The protein precipitate was reconstituted in 8 M urea buffer containing 100 mM sodium phosphate (pH 8.0), 100 mM ammonium bicarbonate, 1% SDS, and 10 mM TCEP and thoroughly sonicated and vortexed to ensure complete resuspension and TCEP reduction. Samples were spun to remove DNA, and the supernatant was transferred to new tubes for alkylation with 20 mM iodoacetamide for 25 min. Following alkylation, 50 mM dithiothreitol was added to the samples, which were then diluted to 4 M urea and 0.5% SDS. Biotinylated proteins were enriched with streptavidin pulldown using equilibrated streptavidin magnetic beads (Pierce) incubated with protein samples overnight at 4 °C. The beads were then washed 3x with wash buffer (4 M urea, 100 mM sodium phosphate [pH 8]) with 0.5% SDS, and 3x with wash buffer without SDS. The beads were subsequently subjected to on-bead digestion with LysC and trypsin. Peptides were then transferred to a new tube, acidified with formic acid (final concentration 3% v/v), desalted, and labeled with TMT reagents as previously described. After TMT labeling, the labeled samples were combined and fractionated using a high-pH reversed-phase peptide fractionation kit (Pierce). The resulting six fractions and an unfractionated sample were analyzed by LC-MS3 as described above.

### Dendra2 half-life determination

HEK 293 cells were plated at 90% confluency in 12-well plates and transfected with Dendra2 constructs using Lipofectamine 2000 (Thermo Fisher) and Opti-Mem medium (Thermo Fisher). After 24 h, cells expressing each construct were transferred into seven wells of flat-bottom 96-well plates for each timepoint of flow cytometry testing. After cells had settled overnight, each well was photoconverted using a fluorescent microscope with a 100-W halogen bulb. After rapid selection of an optimal field of view, one field of view was photoconverted with continual illumination by blue laser for 1 min. Directly thereafter, the next construct was illuminated and photoconverted. Immediately after photoconversion of each timepoint, the sample was rapidly transferred to a round-bottom plate and run on an LSRII flow cytometer (BD Biosciences) with HTS system for data collection. Photoconverted red fluorescent protein positive (RFP^+^) cells were gated from CFP+ singlets against background green fluorescence, which required compensation. After the RFP^+^ population was gated, red fluorescent protein/CFP for the RFP^+^ population was calculated for each construct at each timepoint and plotted.

### Statistics

For all experiments, statistical significance was measured with either a paired or unpaired Student's *t* test (specifics are included in figure legends) with a cutoff of ∗*p* < 0.05, ∗∗*p* < 0.01, ∗∗∗*p* < 0.001, n.s. not significant.

## Data availability

The MS proteomics data have been deposited to the ProteomeXchange Consortium *via* the PRIDE (134) partner repository with the dataset identifier PXD021991 and 10.6019/PXD021991.

## Conflict of interest

S. D., M. W., H. N., A. E., and E. J. B. are current employees of Genentech, Inc. A. M. W. is a former employee of Genentech, Inc. D. F. is a consultant for Genentech, Inc. All the other authors declare that they have no conflicts of interest with the contents of this article.

## References

[1] Blokhuis A.M., Groen E.J., Koppers M., van den Berg L.H., Pasterkamp R.J. (2013). Protein aggregation in amyotrophic lateral sclerosis. Acta Neuropathol..

[2] Ross C.A., Poirier M.A. (2004). Protein aggregation and neurodegenerative disease. Nat. Med..

[3] Wood J.D., Beaujeux T.P., Shaw P.J. (2003). Protein aggregation in motor neurone disorders. Neuropathol. Appl. Neurobiol..

[4] Sreedharan J., Blair I.P., Tripathi V.B., Hu X., Vance C., Rogelj B., Ackerley S., Durnall J.C., Williams K.L., Buratti E., Baralle F., de Belleroche J., Mitchell J.D., Leigh P.N., Al-Chalabi A. (2008). TDP-43 mutations in familial and sporadic amyotrophic lateral sclerosis. Science.

[5] Fecto F., Yan J., Vemula S.P., Liu E., Yang Y., Chen W., Zheng J.G., Shi Y., Siddique N., Arrat H., Donkervoort S., Ajroud-Driss S., Sufit R.L., Heller S.L., Deng H.X. (2011). SQSTM1 mutations in familial and sporadic amyotrophic lateral sclerosis. Arch. Neurol..

[6] Maruyama H., Morino H., Ito H., Izumi Y., Kato H., Watanabe Y., Kinoshita Y., Kamada M., Nodera H., Suzuki H., Komure O., Matsuura S., Kobatake K., Morimoto N., Abe K. (2010). Mutations of optineurin in amyotrophic lateral sclerosis. Nature.

[7] Kiernan M.C., Vucic S., Cheah B.C., Turner M.R., Eisen A., Hardiman O., Burrell J.R., Zoing M.C. (2011). Amyotrophic lateral sclerosis. Lancet.

[8] Chen X., Ebelle D.L., Wright B.J., Sridharan V., Hooper E., Walters K.J. (2019). Structure of hRpn10 bound to UBQLN2 UBL illustrates basis for complementarity between shuttle factors and substrates at the proteasome. J. Mol. Biol..

[9] Kleijnen M.F., Alarcon R.M., Howley P.M. (2003). The ubiquitin-associated domain of hPLIC-2 interacts with the proteasome. Mol. Biol. Cell.

[10] Kleijnen M.F., Shih A.H., Zhou P., Kumar S., Soccio R.E., Kedersha N.L., Gill G., Howley P.M. (2000). The hPLIC proteins may provide a link between the ubiquitination machinery and the proteasome. Mol. Cell.

[11] Zhang D., Raasi S., Fushman D. (2008). Affinity makes the difference: nonselective interaction of the UBA domain of Ubiquilin-1 with monomeric ubiquitin and polyubiquitin chains. J. Mol. Biol..

[12] Biggins S., Ivanovska I., Rose M.D. (1996). Yeast ubiquitin-like genes are involved in duplication of the microtubule organizing center. J. Cell Biol..

[13] Elsasser S., Finley D. (2005). Delivery of ubiquitinated substrates to protein-unfolding machines. Nat. Cell Biol.

[14] Liu C., van Dyk D., Li Y., Andrews B., Rao H. (2009). A genome-wide synthetic dosage lethality screen reveals multiple pathways that require the functioning of ubiquitin-binding proteins Rad23 and Dsk2. BMC Biol..

[15] Medicherla B., Kostova Z., Schaefer A., Wolf D.H. (2004). A genomic screen identifies Dsk2p and Rad23p as essential components of ER-associated degradation. EMBO Rep..

[16] Elsasser S., Gali R.R., Schwickart M., Larsen C.N., Leggett D.S., Muller B., Feng M.T., Tubing F., Dittmar G.A., Finley D. (2002). Proteasome subunit Rpn1 binds ubiquitin-like protein domains. Nat. Cell Biol..

[17] Finley D. (2009). Recognition and processing of ubiquitin-protein conjugates by the proteasome. Annu. Rev. Biochem..

[18] Walters K.J., Kleijnen M.F., Goh A.M., Wagner G., Howley P.M. (2002). Structural studies of the interaction between ubiquitin family proteins and proteasome subunit S5a. Biochemistry.

[19] Shi Y., Chen X., Elsasser S., Stocks B.B., Tian G., Lee B.H., Shi Y., Zhang N., de Poot S.A., Tuebing F., Sun S., Vannoy J., Tarasov S.G., Engen J.R., Finley D. (2016). Rpn1 provides adjacent receptor sites for substrate binding and deubiquitination by the proteasome. Science.

[20] Chen X., Randles L., Shi K., Tarasov S.G., Aihara H., Walters K.J. (2016). Structures of Rpn1 T1:Rad23 and hRpn13:hPLIC2 reveal distinct binding mechanisms between substrate receptors and shuttle factors of the proteasome. Structure.

[21] Rao H., Sastry A. (2002). Recognition of specific ubiquitin conjugates is important for the proteolytic functions of the ubiquitin-associated domain proteins Dsk2 and Rad23. J. Biol. Chem..

[22] Saeki Y., Saitoh A., Toh-e A., Yokosawa H. (2002). Ubiquitin-like proteins and Rpn10 play cooperative roles in ubiquitin-dependent proteolysis. Biochem. Biophys. Res. Commun..

[23] Wilkinson C.R., Seeger M., Hartmann-Petersen R., Stone M., Wallace M., Semple C., Gordon C. (2001). Proteins containing the UBA domain are able to bind to multi-ubiquitin chains. Nat. Cell Biol..

[24] Funakoshi M., Sasaki T., Nishimoto T., Kobayashi H. (2002). Budding yeast Dsk2p is a polyubiquitin-binding protein that can interact with the proteasome. Proc. Natl. Acad. Sci. U. S. A..

[25] Harman C.A., Monteiro M.J. (2019). The specificity of ubiquitin binding to ubiquilin-1 is regulated by sequences besides its UBA domain. Biochim. Biophys. Acta Gen. Subj..

[26] Hjerpe R., Aillet F., Lopitz-Otsoa F., Lang V., England P., Rodriguez M.S. (2009). Efficient protection and isolation of ubiquitylated proteins using tandem ubiquitin-binding entities. EMBO Rep..

[27] Ko H.S., Uehara T., Tsuruma K., Nomura Y. (2004). Ubiquilin interacts with ubiquitylated proteins and proteasome through its ubiquitin-associated and ubiquitin-like domains. FEBS Lett..

[28] Sims J.J., Haririnia A., Dickinson B.C., Fushman D., Cohen R.E. (2009). Avid interactions underlie the Lys63-linked polyubiquitin binding specificities observed for UBA domains. Nat. Struct. Mol. Biol..

[29] Dao T.P., Kolaitis R.M., Kim H.J., O'Donovan K., Martyniak B., Colicino E., Hehnly H., Taylor J.P., Castaneda C.A. (2018). Ubiquitin modulates liquid-liquid phase separation of UBQLN2 via disruption of multivalent interactions. Mol. Cell.

[30] Itakura E., Zavodszky E., Shao S., Wohlever M.L., Keenan R.J., Hegde R.S. (2016). Ubiquilins chaperone and triage mitochondrial membrane proteins for degradation. Mol. Cell.

[31] Tsuchiya H., Ohtake F., Arai N., Kaiho A., Yasuda S., Tanaka K., Saeki Y. (2017). In vivo ubiquitin linkage-type analysis reveals that the Cdc48-Rad23/Dsk2 Axis contributes to K48-linked chain specificity of the proteasome. Mol. Cell.

[32] Suzuki R., Kawahara H. (2016). UBQLN4 recognizes mislocalized transmembrane domain proteins and targets these to proteasomal degradation. EMBO Rep..

[33] Hjerpe R., Bett J.S., Keuss M.J., Solovyova A., McWilliams T.G., Johnson C., Sahu I., Varghese J., Wood N., Wightman M., Osborne G., Bates G.P., Glickman M.H., Trost M., Knebel A. (2016). UBQLN2 mediates autophagy-independent protein aggregate clearance by the proteasome. Cell.

[34] Whiteley A.M., Prado M.A., Peng I., Abbas A.R., Haley B., Paulo J.A., Reichelt M., Katakam A., Sagolla M., Modrusan Z., Lee D.Y., Roose-Girma M., Kirkpatrick D.S., McKenzie B.S., Gygi S.P. (2017). Ubiquilin1 promotes antigen-receptor mediated proliferation by eliminating mislocalized mitochondrial proteins. Elife.

[35] Marin I. (2014). The ubiquilin gene family: evolutionary patterns and functional insights. BMC Evol. Biol..

[36] Lee D.Y., Arnott D., Brown E.J. (2013). Ubiquilin4 is an adaptor protein that recruits Ubiquilin1 to the autophagy machinery. EMBO Rep..

[37] N'Diaye E.N., Debnath J., Brown E.J. (2009). Ubiquilins accelerate autophagosome maturation and promote cell survival during nutrient starvation. Autophagy.

[38] N'Diaye E.N., Kajihara K.K., Hsieh I., Morisaki H., Debnath J., Brown E.J. (2009). PLIC proteins or ubiquilins regulate autophagy-dependent cell survival during nutrient starvation. EMBO Rep..

[39] Rothenberg C., Srinivasan D., Mah L., Kaushik S., Peterhoff C.M., Ugolino J., Fang S., Cuervo A.M., Nixon R.A., Monteiro M.J. (2010). Ubiquilin functions in autophagy and is degraded by chaperone-mediated autophagy. Hum. Mol. Genet..

[40] Senturk M., Lin G., Zuo Z., Mao D., Watson E., Mikos A.G., Bellen H.J. (2019). Ubiquilins regulate autophagic flux through mTOR signalling and lysosomal acidification. Nat. Cell Biol..

[41] Wu J.J., Cai A., Greenslade J.E., Higgins N.R., Fan C., Le N.T.T., Tatman M., Whiteley A.M., Prado M.A., Dieriks B.V., Curtis M.A., Shaw C.E., Siddique T., Faull R.L.M., Scotter E.L. (2020). ALS/FTD mutations in UBQLN2 impede autophagy by reducing autophagosome acidification through loss of function. Proc. Natl. Acad. Sci. U. S. A..

[42] Gilpin K.M., Chang L., Monteiro M.J. (2015). ALS-linked mutations in ubiquilin-2 or hnRNPA1 reduce interaction between ubiquilin-2 and hnRNPA1. Hum. Mol. Genet..

[43] Chang L., Monteiro M.J. (2015). Defective proteasome delivery of polyubiquitinated proteins by ubiquilin-2 proteins containing ALS mutations. PLoS One.

[44] Alexander E.J., Ghanbari Niaki A., Zhang T., Sarkar J., Liu Y., Nirujogi R.S., Pandey A., Myong S., Wang J. (2018). Ubiquilin 2 modulates ALS/FTD-linked FUS-RNA complex dynamics and stress granule formation. Proc. Natl. Acad. Sci. U. S. A..

[45] Sharkey L.M., Safren N., Pithadia A.S., Gerson J.E., Dulchavsky M., Fischer S., Patel R., Lantis G., Ashraf N., Kim J.H., Meliki A., Minakawa E.N., Barmada S.J., Ivanova M.I., Paulson H.L. (2018). Mutant UBQLN2 promotes toxicity by modulating intrinsic self-assembly. Proc. Natl. Acad. Sci. U. S. A..

[46] Sharkey L.M., Sandoval-Pistorius S.S., Moore S.J., Gerson J.E., Komlo R., Fischer S., Negron-Rios K.Y., Crowley E.V., Padron F., Patel R., Murphy G.G., Paulson H.L. (2020). Modeling UBQLN2-mediated neurodegenerative disease in mice: shared and divergent properties of wild type and mutant UBQLN2 in phase separation, subcellular localization, altered proteostasis pathways, and selective cytotoxicity. Neurobiol. Dis..

[47] Deng H.X., Chen W., Hong S.T., Boycott K.M., Gorrie G.H., Siddique N., Yang Y., Fecto F., Shi Y., Zhai H., Jiang H., Hirano M., Rampersaud E., Jansen G.H., Donkervoort S. (2011). Mutations in UBQLN2 cause dominant X-linked juvenile and adult-onset ALS and ALS/dementia. Nature.

[48] Gerson J.E., Safren N., Fischer S., Patel R., Crowley E.V., Welday J.P., Windle A.K., Barmada S., Paulson H.L., Sharkey L.M. (2020). Ubiquilin-2 differentially regulates polyglutamine disease proteins. Hum. Mol. Genet..

[49] Wu Q., Liu M., Huang C., Liu X., Huang B., Li N., Zhou H., Xia X.G. (2015). Pathogenic Ubqln2 gains toxic properties to induce neuron death. Acta Neuropathol..

[50] Teyssou E., Chartier L., Amador M.D., Lam R., Lautrette G., Nicol M., Machat S., Da Barroca S., Moigneu C., Mairey M., Larmonier T., Saker S., Dussert C., Forlani S., Fontaine B. (2017). Novel UBQLN2 mutations linked to amyotrophic lateral sclerosis and atypical hereditary spastic paraplegia phenotype through defective HSP70-mediated proteolysis. Neurobiol. Aging.

[51] Williams K.L., Warraich S.T., Yang S., Solski J.A., Fernando R., Rouleau G.A., Nicholson G.A., Blair I.P. (2012). UBQLN2/ubiquilin 2 mutation and pathology in familial amyotrophic lateral sclerosis. Neurobiol. Aging.

[52] Daoud H., Suhail H., Szuto A., Camu W., Salachas F., Meininger V., Bouchard J.P., Dupre N., Dion P.A., Rouleau G.A. (2012). UBQLN2 mutations are rare in French and French-Canadian amyotrophic lateral sclerosis. Neurobiol. Aging.

[53] Synofzik M., Maetzler W., Grehl T., Prudlo J., Vom Hagen J.M., Haack T., Rebassoo P., Munz M., Schols L., Biskup S. (2012). Screening in ALS and FTD patients reveals 3 novel UBQLN2 mutations outside the PXX domain and a pure FTD phenotype. Neurobiol. Aging.

[54] Rutherford N.J., Lewis J., Clippinger A.K., Thomas M.A., Adamson J., Cruz P.E., Cannon A., Xu G., Golde T.E., Shaw G., Borchelt D.R., Giasson B.I. (2013). Unbiased screen reveals ubiquilin-1 and -2 highly associated with huntingtin inclusions. Brain Res..

[55] Wang H., Lim P.J., Yin C., Rieckher M., Vogel B.E., Monteiro M.J. (2006). Suppression of polyglutamine-induced toxicity in cell and animal models of Huntington's disease by ubiquilin. Hum. Mol. Genet..

[56] El Ayadi A., Stieren E.S., Barral J.M., Boehning D. (2012). Ubiquilin-1 regulates amyloid precursor protein maturation and degradation by stimulating K63-linked polyubiquitination of lysine 688. Proc. Natl. Acad. Sci. U. S. A..

[57] Mah A.L., Perry G., Smith M.A., Monteiro M.J. (2000). Identification of ubiquilin, a novel presenilin interactor that increases presenilin protein accumulation. J. Cell Biol..

[58] Ford D.L., Monteiro M.J. (2006). Dimerization of ubiquilin is dependent upon the central region of the protein: evidence that the monomer, but not the dimer, is involved in binding presenilins. Biochem. J..

[59] Le N.T., Chang L., Kovlyagina I., Georgiou P., Safren N., Braunstein K.E., Kvarta M.D., Van Dyke A.M., LeGates T.A., Philips T., Morrison B.M., Thompson S.M., Puche A.C., Gould T.D., Rothstein J.D. (2016). Motor neuron disease, TDP-43 pathology, and memory deficits in mice expressing ALS-FTD-linked UBQLN2 mutations. Proc. Natl. Acad. Sci. U. S. A..

[60] Lomash R.M., Gu X., Youle R.J., Lu W., Roche K.W. (2015). Neurolastin, a dynamin family GTPase, regulates excitatory synapses and spine density. Cell Rep..

[61] Sato T., Okumura F., Kano S., Kondo T., Ariga T., Hatakeyama S. (2011). TRIM32 promotes neural differentiation through retinoic acid receptor-mediated transcription. J. Cell Sci.

[62] Chiang A.P., Beck J.S., Yen H.J., Tayeh M.K., Scheetz T.E., Swiderski R.E., Nishimura D.Y., Braun T.A., Kim K.Y., Huang J., Elbedour K., Carmi R., Slusarski D.C., Casavant T.L., Stone E.M. (2006). Homozygosity mapping with SNP arrays identifies TRIM32, an E3 ubiquitin ligase, as a Bardet-Biedl syndrome gene (BBS11). Proc. Natl. Acad. Sci. U. S. A..

[63] Kudryashova E., Kudryashov D., Kramerova I., Spencer M.J. (2005). Trim32 is a ubiquitin ligase mutated in limb girdle muscular dystrophy type 2H that binds to skeletal muscle myosin and ubiquitinates actin. J. Mol. Biol..

[64] Locke M., Tinsley C.L., Benson M.A., Blake D.J. (2009). TRIM32 is an E3 ubiquitin ligase for dysbindin. Hum. Mol. Genet..

[65] Cohen S., Zhai B., Gygi S.P., Goldberg A.L. (2012). Ubiquitylation by Trim32 causes coupled loss of desmin, Z-bands, and thin filaments in muscle atrophy. J. Cell Biol..

[66] Zhang J., Hu M.M., Wang Y.Y., Shu H.B. (2012). TRIM32 protein modulates type I interferon induction and cellular antiviral response by targeting MITA/STING protein for K63-linked ubiquitination. J. Biol. Chem..

[67] Lee Y.C., Huang W.C., Lin J.H., Kao T.J., Lin H.C., Lee K.H., Lin H.C., Shen C.J., Chang W.C., Huang C.C. (2018). Znf179 E3 ligase-mediated TDP-43 polyubiquitination is involved in TDP-43- ubiquitinated inclusions (UBI) (+)-related neurodegenerative pathology. J. Biomed. Sci..

[68] Chen Y.C., Umanah G.K., Dephoure N., Andrabi S.A., Gygi S.P., Dawson T.M., Dawson V.L., Rutter J. (2014). Msp1/ATAD1 maintains mitochondrial function by facilitating the degradation of mislocalized tail-anchored proteins. EMBO J..

[69] Okreglak V., Walter P. (2014). The conserved AAA-ATPase Msp1 confers organelle specificity to tail-anchored proteins. Proc. Natl. Acad. Sci. U. S. A..

[70] Brandt J., Veith A.M., Volff J.N. (2005). A family of neofunctionalized Ty3/gypsy retrotransposon genes in mammalian genomes. Cytogenet. Genome Res..

[71] Lux A., Beil C., Majety M., Barron S., Gallione C.J., Kuhn H.-M., Berg J.N., Kioschis P., Marchuk D.A., Hafner M. (2005). Human retroviral gag - and gag-pol -like proteins interact with the transforming growth factor-β receptor activin receptor-like kinase 1. J. Biol. Chem.

[72] Lux H., Flammann H., Hafner M., Lux A. (2010). Genetic and molecular analyses of PEG10 reveal new aspects of genomic organization, transcription and translation. PLoS ONE.

[73] Clark M.B., Janicke M., Gottesbuhren U., Kleffmann T., Legge M., Poole E.S., Tate W.P. (2007). Mammalian gene PEG10 expresses two reading frames by high efficiency -1 frameshifting in embryonic-associated tissues. J. Biol. Chem..

[74] Shigemoto K., Brennan J., Walls E., Watson C.J., Stott D., Rigby P.W., Reith A.D. (2001). Identification and characterisation of a developmentally regulated mammalian gene that utilises -1 programmed ribosomal frameshifting. Nucleic Acids Res..

[75] Abed M., Verschueren E., Budayeva H., Liu P., Kirkpatrick D.S., Reja R., Kummerfeld S.K., Webster J.D., Gierke S., Reichelt M., Anderson K.R., Newman R.J., Roose-Girma M., Modrusan Z., Pektas H. (2019). The Gag protein PEG10 binds to RNA and regulates trophoblast stem cell lineage specification. PLoS One.

[76] Akamatsu S., Wyatt A.W., Lin D., Lysakowski S., Zhang F., Kim S., Tse C., Wang K., Mo F., Haegert A., Brahmbhatt S., Bell R., Adomat H., Kawai Y., Xue H. (2015). The placental gene PEG10 promotes progression of neuroendocrine prostate cancer. Cell Rep..

[77] Ono R., Nakamura K., Inoue K., Naruse M., Usami T., Wakisaka-Saito N., Hino T., Suzuki-Migishima R., Ogonuki N., Miki H., Kohda T., Ogura A., Yokoyama M., Kaneko-Ishino T., Ishino F. (2006). Deletion of Peg10, an imprinted gene acquired from a retrotransposon, causes early embryonic lethality. Nat. Genet..

[78] Ashley J., Cordy B., Lucia D., Fradkin L.G., Budnik V., Thomson T. (2018). Retrovirus-like gag protein Arc1 binds RNA and traffics across synaptic boutons. Cell.

[79] Pastuzyn E.D., Day C.E., Kearns R.B., Kyrke-Smith M., Taibi A.V., McCormick J., Yoder N., Belnap D.M., Erlendsson S., Morado D.R., Briggs J.A.G., Feschotte C., Shepherd J.D. (2018). The neuronal gene arc encodes a repurposed retrotransposon gag protein that mediates intercellular RNA transfer. Cell.

[80] Zhang Y., Pak C., Han Y., Ahlenius H., Zhang Z., Chanda S., Marro S., Patzke C., Acuna C., Covy J., Xu W., Yang N., Danko T., Chen L., Wernig M. (2013). Rapid single-step induction of functional neurons from human pluripotent stem cells. Neuron.

[81] N'Diaye E.N., Hanyaloglu A.C., Kajihara K.K., Puthenveedu M.A., Wu P., von Zastrow M., Brown E.J. (2008). The ubiquitin-like protein PLIC-2 is a negative regulator of G protein-coupled receptor endocytosis. Mol. Biol. Cell.

[82] Kaksonen M., Roux A. (2018). Mechanisms of clathrin-mediated endocytosis. Nat. Rev. Mol. Cell Biol..

[83] Komander D., Rape M. (2012). The ubiquitin code. Annu. Rev. Biochem..

[84] Lauwers E., Jacob C., Andre B. (2009). K63-linked ubiquitin chains as a specific signal for protein sorting into the multivesicular body pathway. J. Cell Biol..

[85] Fritsch J., Stephan M., Tchikov V., Winoto-Morbach S., Gubkina S., Kabelitz D., Schutze S. (2014). Cell fate decisions regulated by K63 ubiquitination of tumor necrosis factor receptor 1. Mol. Cell Biol..

[86] Lam S.S., Martell J.D., Kamer K.J., Deerinck T.J., Ellisman M.H., Mootha V.K., Ting A.Y. (2015). Directed evolution of APEX2 for electron microscopy and proximity labeling. Nat. Methods.

[87] Kurlawala Z., Saurabh K., Dunaway R., Shah P.P., Siskind L.J., Beverly L.J. (2020). Ubiquilin proteins regulate EGFR levels and activity in lung adenocarcinoma cells. J. Cell Biochem..

[88] Blokhuis A.M., Koppers M., Groen E.J.N., van den Heuvel D.M.A., Dini Modigliani S., Anink J.J., Fumoto K., van Diggelen F., Snelting A., Sodaar P., Verheijen B.M., Demmers J.A.A., Veldink J.H., Aronica E., Bozzoni I. (2016). Comparative interactomics analysis of different ALS-associated proteins identifies converging molecular pathways. Acta Neuropathol..

[89] Szklarczyk D., Gable A.L., Lyon D., Junge A., Wyder S., Huerta-Cepas J., Simonovic M., Doncheva N.T., Morris J.H., Bork P., Jensen L.J., Mering C.V. (2019). STRING v11: protein-protein association networks with increased coverage, supporting functional discovery in genome-wide experimental datasets. Nucleic Acids Res..

[90] Schmidt M.H.H., Dikic I. (2005). The Cbl interactome and its functions. Nat. Rev. Mol. Cell Biol..

[91] Sigismund S., Algisi V., Nappo G., Conte A., Pascolutti R., Cuomo A., Bonaldi T., Argenzio E., Verhoef L.G., Maspero E., Bianchi F., Capuani F., Ciliberto A., Polo S., Di Fiore P.P. (2013). Threshold-controlled ubiquitination of the EGFR directs receptor fate. EMBO J..

[92] Goh L.K., Sorkin A. (2013). Endocytosis of receptor tyrosine kinases. Cold Spring Harb. Perspect. Biol..

[93] Piper R.C., Dikic I., Lukacs G.L. (2014). Ubiquitin-dependent sorting in endocytosis. Cold Spring Harb. Perspect. Biol..

[94] Vietri M., Radulovic M., Stenmark H. (2020). The many functions of ESCRTs. Nat. Rev. Mol. Cell Biol.

[95] Fritsch J., Zingler P., Sarchen V., Heck A.L., Schutze S. (2017). Role of ubiquitination and proteolysis in the regulation of pro- and anti-apoptotic TNF-R1 signaling. Biochim. Biophys. Acta Mol. Cell Res.

[96] Zhang L., Zhou F., Garcia de Vinuesa A., de Kruijf E.M., Mesker W.E., Hui L., Drabsch Y., Li Y., Bauer A., Rousseau A., Sheppard K.A., Mickanin C., Kuppen P.J., Lu C.X., Ten Dijke P. (2013). TRAF4 promotes TGF-beta receptor signaling and drives breast cancer metastasis. Mol. Cell.

[97] Blaise S., Kneib M., Rousseau A., Gambino F., Chenard M.P., Messadeq N., Muckenstrum M., Alpy F., Tomasetto C., Humeau Y., Rio M.C. (2012). In vivo evidence that TRAF4 is required for central nervous system myelin homeostasis. PLoS One.

[98] Maminska A., Bartosik A., Banach-Orlowska M., Pilecka I., Jastrzebski K., Zdzalik-Bielecka D., Castanon I., Poulain M., Neyen C., Wolinska-Niziol L., Torun A., Szymanska E., Kowalczyk A., Piwocka K., Simonsen A. (2016). ESCRT proteins restrict constitutive NF-kappaB signaling by trafficking cytokine receptors. Sci. Signal.

[99] Shi J.H., Sun S.C. (2018). Tumor necrosis factor receptor-associated factor regulation of nuclear factor kappaB and mitogen-activated protein kinase pathways. Front. Immunol..

[100] Yan J., Li Q., Mao A.P., Hu M.M., Shu H.B. (2014). TRIM4 modulates type I interferon induction and cellular antiviral response by targeting RIG-I for K63-linked ubiquitination. J. Mol. Cell Biol.

[101] Fletcher A.J., Vaysburd M., Maslen S., Zeng J., Skehel J.M., Towers G.J., James L.C. (2018). Trivalent RING assembly on retroviral capsids activates TRIM5 ubiquitination and innate immune signaling. Cell Host Microbe.

[102] Davis M.E., Gack M.U. (2015). Ubiquitination in the antiviral immune response. Virology.

[103] Liu C., Huang S., Wang X., Wen M., Zheng J., Wang W., Fu Y., Tian S., Li L., Li Z., Wang X. (2019). The otubain YOD1 suppresses aggregation and activation of the signaling adaptor MAVS through Lys63-linked deubiquitination. J. Immunol..

[104] Bjorkoy G., Lamark T., Brech A., Outzen H., Perander M., Overvatn A., Stenmark H., Johansen T. (2005). p62/SQSTM1 forms protein aggregates degraded by autophagy and has a protective effect on huntingtin-induced cell death. J. Cell Biol.

[105] Kirkin V., Lamark T., Sou Y.S., Bjorkoy G., Nunn J.L., Bruun J.A., Shvets E., McEwan D.G., Clausen T.H., Wild P., Bilusic I., Theurillat J.P., Overvatn A., Ishii T., Elazar Z. (2009). A role for NBR1 in autophagosomal degradation of ubiquitinated substrates. Mol. Cell.

[106] Grumati P., Dikic I. (2018). Ubiquitin signaling and autophagy. J. Biol. Chem..

[107] Shin E.J., Shin H.M., Nam E., Kim W.S., Kim J.H., Oh B.H., Yun Y. (2012). DeSUMOylating isopeptidase: a second class of SUMO protease. EMBO Rep..

[108] Hirayama S., Sugihara M., Morito D., Iemura S.I., Natsume T., Murata S., Nagata K. (2018). Nuclear export of ubiquitinated proteins via the UBIN-POST system. Proc. Natl. Acad. Sci. U. S. A..

[109] Huttlin E.L., Bruckner R.J., Paulo J.A., Cannon J.R., Ting L., Baltier K., Colby G., Gebreab F., Gygi M.P., Parzen H., Szpyt J., Tam S., Zarraga G., Pontano-Vaites L., Swarup S. (2017). Architecture of the human interactome defines protein communities and disease networks. Nature.

[110] Schweppe D.K., Huttlin E.L., Harper J.W., Gygi S.P. (2018). BioPlex display: an interactive suite for large-scale AP-MS protein-protein interaction data. J. Proteome Res..

[111] Rose C.M., Isasa M., Ordureau A., Prado M.A., Beausoleil S.A., Jedrychowski M.P., Finley D.J., Harper J.W., Gygi S.P. (2016). Highly multiplexed quantitative mass spectrometry analysis of ubiquitylomes. Cell Syst..

[112] Massey L.K., Mah A.L., Ford D.L., Miller J., Liang J., Doong H., Monteiro M.J. (2004). Overexpression of ubiquilin decreases ubiquitination and degradation of presenilin proteins. J. Alzheimers Dis..

[113] Gurskaya N.G., Verkhusha V.V., Shcheglov A.S., Staroverov D.B., Chepurnykh T.V., Fradkov A.F., Lukyanov S., Lukyanov K.A. (2006). Engineering of a monomeric green-to-red photoactivatable fluorescent protein induced by blue light. Nat. Biotechnol..

[114] Halloran M., Ragagnin A.M.G., Vidal M., Parakh S., Yang S., Heng B., Grima N., Shahheydari H., Soo K.Y., Blair I., Guillemin G.J., Sundaramoorthy V., Atkin J.D. (2020). Amyotrophic lateral sclerosis-linked UBQLN2 mutants inhibit endoplasmic reticulum to Golgi transport, leading to Golgi fragmentation and ER stress. Cell Mol. Life Sci..

[115] Inoue M., Takahashi K., Niide O., Shibata M., Fukuzawa M., Ra C. (2005). LDOC1, a novel MZF-1-interacting protein, induces apoptosis. FEBS Lett..

[116] Nagasaki K., Schem C., von Kaisenberg C., Biallek M., Rosel F., Jonat W., Maass N. (2003). Leucine-zipper protein, LDOC1, inhibits NF-kappaB activation and sensitizes pancreatic cancer cells to apoptosis. Int. J. Cancer.

[117] Yokota T., Mishra M., Akatsu H., Tani Y., Miyauchi T., Yamamoto T., Kosaka K., Nagai Y., Sawada T., Heese K. (2006). Brain site-specific gene expression analysis in Alzheimer's disease patients. Eur. J. Clin. Invest.

[118] Assereto S., Piccirillo R., Baratto S., Scudieri P., Fiorillo C., Massacesi M., Traverso M., Galietta L.J., Bruno C., Minetti C., Zara F., Gazzerro E. (2016). The ubiquitin ligase tripartite-motif-protein 32 is induced in Duchenne muscular dystrophy. Lab. Invest.

[119] Liu Y., Wu W., Yang H., Zhou Z., Zhu X., Sun C., Liu Y., Yu Z., Chen Y., Wang Y. (2017). Upregulated expression of TRIM32 is involved in Schwann cell differentiation, migration and neurite outgrowth after sciatic nerve crush. Neurochem. Res..

[120] Vermeiren Y., Janssens J., Van Dam D., De Deyn P.P. (2018). Serotonergic dysfunction in amyotrophic lateral sclerosis and Parkinson's disease: similar mechanisms, dissimilar outcomes. Front. Neurosci..

[121] Chen G., Gulbranson D.R., Hou Z., Bolin J.M., Ruotti V., Probasco M.D., Smuga-Otto K., Howden S.E., Diol N.R., Propson N.E., Wagner R., Lee G.O., Antosiewicz-Bourget J., Teng J.M., Thomson J.A. (2011). Chemically defined conditions for human iPSC derivation and culture. Nat. Methods.

[122] Zuris J.A., Thompson D.B., Shu Y., Guilinger J.P., Bessen J.L., Hu J.H., Maeder M.L., Joung J.K., Chen Z.Y., Liu D.R. (2015). Cationic lipid-mediated delivery of proteins enables efficient protein-based genome editing in vitro and in vivo. Nat. Biotechnol..

[123] Guyenet S.J., Furrer S.A., Damian V.M., Baughan T.D., La Spada A.R., Garden G.A. (2010). A simple composite phenotype scoring system for evaluating mouse models of cerebellar ataxia. J. Vis. Exp..

[124] Weber M., Wu T., Hanson J.E., Alam N.M., Solanoy H., Ngu H., Lauffer B.E., Lin H.H., Dominguez S.L., Reeder J., Tom J., Steiner P., Foreman O., Prusky G.T., Scearce-Levie K. (2015). Cognitive deficits, changes in synaptic function, and brain pathology in a mouse model of normal aging(1,2,3). eNeuro.

[125] Foldi C.J., Eyles D.W., McGrath J.J., Burne T.H. (2011). The effects of breeding protocol in C57BL/6J mice on adult offspring behaviour. PLoS One.

[126] McAlister G.C., Nusinow D.P., Jedrychowski M.P., Wuhr M., Huttlin E.L., Erickson B.K., Rad R., Haas W., Gygi S.P. (2014). MultiNotch MS3 enables accurate, sensitive, and multiplexed detection of differential expression across cancer cell line proteomes. Anal. Chem..

[127] Huttlin E.L., Jedrychowski M.P., Elias J.E., Goswami T., Rad R., Beausoleil S.A., Villen J., Haas W., Sowa M.E., Gygi S.P. (2010). A tissue-specific atlas of mouse protein phosphorylation and expression. Cell.

[128] Navarrete-Perea J., Yu Q., Gygi S.P., Paulo J.A. (2018). Streamlined tandem mass tag (SL-TMT) protocol: an efficient strategy for quantitative (phospho)proteome profiling using tandem mass tag-synchronous precursor selection-MS3. J. Proteome Res..

[129] Paulo J.A., O'Connell J.D., Gygi S.P. (2016). A triple knockout (TKO) proteomics standard for diagnosing ion interference in isobaric labeling experiments. J. Am. Soc. Mass Spectrom..

[130] Yu G., Wang L.G., Han Y., He Q.Y. (2012). clusterProfiler: an R package for comparing biological themes among gene clusters. OMICS.

[131] Le Pichon C.E., Dominguez S.L., Solanoy H., Ngu H., Lewin-Koh N., Chen M., Eastham-Anderson J., Watts R., Scearce-Levie K. (2013). EGFR inhibitor erlotinib delays disease progression but does not extend survival in the SOD1 mouse model of ALS. PLoS One.

[132] Kallop D.Y., Meilandt W.J., Gogineni A., Easley-Neal C., Wu T., Jubb A.M., Yaylaoglu M., Shamloo M., Tessier-Lavigne M., Scearce-Levie K., Weimer R.M. (2014). A death receptor 6-amyloid precursor protein pathway regulates synapse density in the mature CNS but does not contribute to Alzheimer's disease-related pathophysiology in murine models. J. Neurosci..

[133] Paek J., Kalocsay M., Staus D.P., Wingler L., Pascolutti R., Paulo J.A., Gygi S.P., Kruse A.C. (2017). Multidimensional tracking of GPCR signaling via peroxidase-catalyzed proximity labeling. Cell.

[134] Perez-Riverol Y., Csordas A., Bai J., Bernal-Llinares M., Hewapathirana S., Kundu D.J., Inuganti A., Griss J., Mayer G., Eisenacher M., Perez E., Uszkoreit J., Pfeuffer J., Sachsenberg T., Yilmaz S. (2019). The PRIDE database and related tools and resources in 2019: improving support for quantification data. Nucleic Acids Res..

