# Supplementary material for: Global proteomics of Ubqln2-based murine models of ALS
Source: J Biol Chem. 2020 Dec 10;296:100153. doi: 10.1074/jbc.RA120.015960 (PMC7873701; doi:10.1074/jbc.RA120.015960)
Supplement: Supplementary file 1 — Supplemental Material [file mmc1.pdf]

Supplemental Figure 1

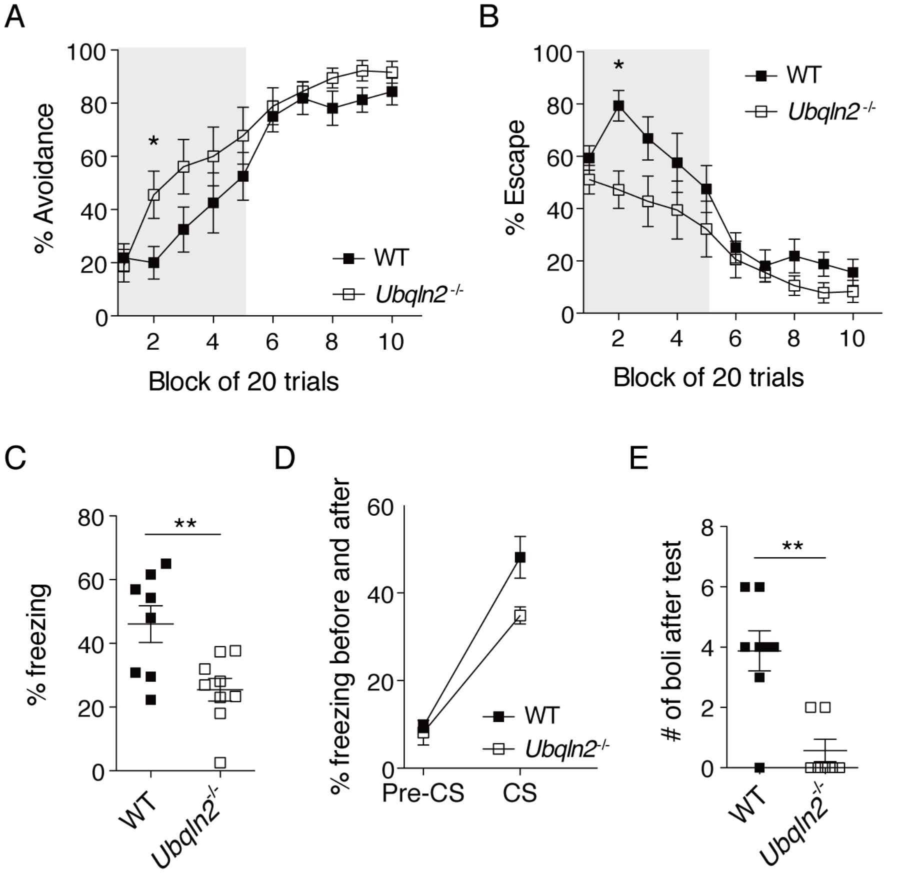

Supplemental Figure 1. *Ubqln2$^{-/-}$* mice have memory deficits.
(A, B) *Ubqln2$^{-/-}$* mice exhibit largely normal active avoidance behavior.
(C) *Ubqln2$^{-/-}$* mice exhibit reduced frequency of freezing during the contextual fear conditioning test.
(D) *Ubqln2$^{-/-}$* mice have a mild decrease in frequency of freezing during presentation of the conditioning stimulus (CS) in a cued fear conditioning test.
(E) The number of fecal boli left in the chamber after the cued-fear condition test period was used as a proxy measure of anxiety and emotionality. *Ubqln2$^{-/-}$* mice had fewer boli than WT controls. Statistical significance was determined with two-tailed, unpaired Student's T test. n=8-9 animals.

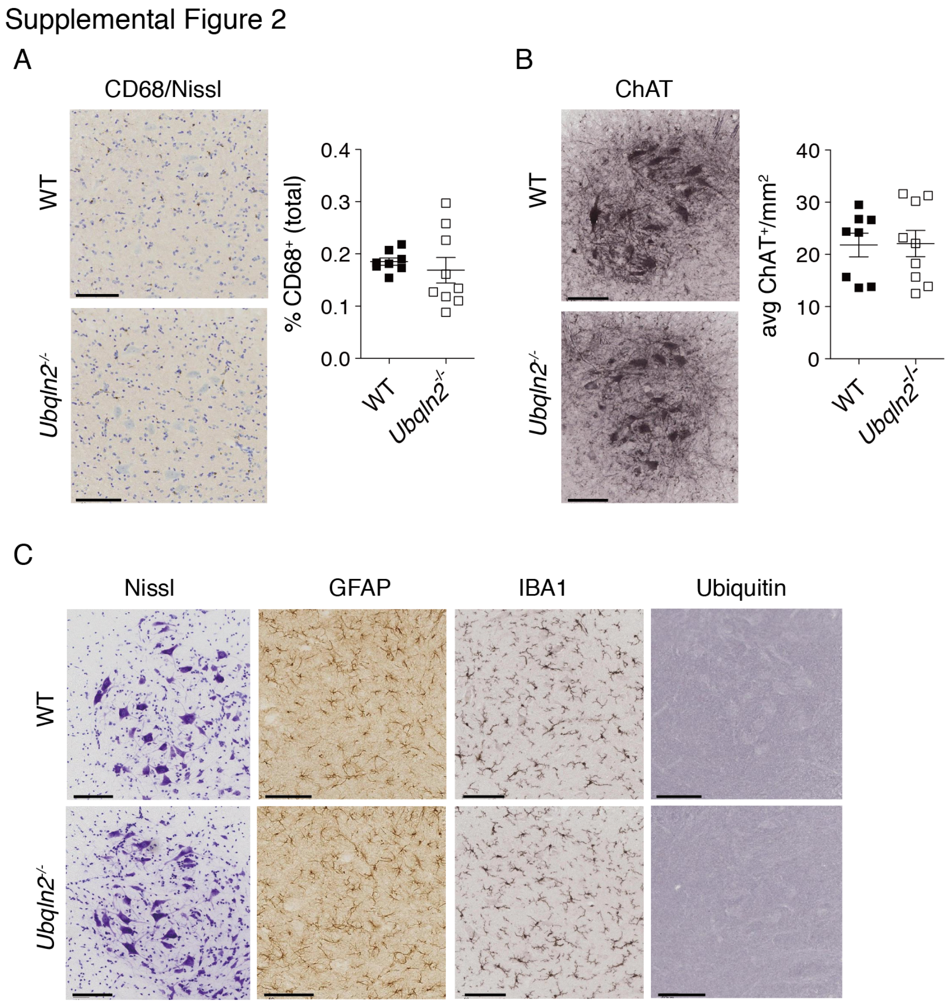

Supplemental Figure 2. Histological staining of *Ubqln2$^{-/-}$* animals.
Spinal cords of approximately one-year-old mice were stained histologically.
(A) CD68 and Nissl co-staining revealed no changes in neuroinflammation.
(B) *Ubqln2$^{-/-}$* spinal cords have normal numbers of ChAT+ neurons.
(C) No significant changes were observed in Nissl, GFAP, IBA1, or Ubiquitin staining. n=8-9 animals per genotype. Scale bar = 100 µm for each image.

Supplemental Figure 3

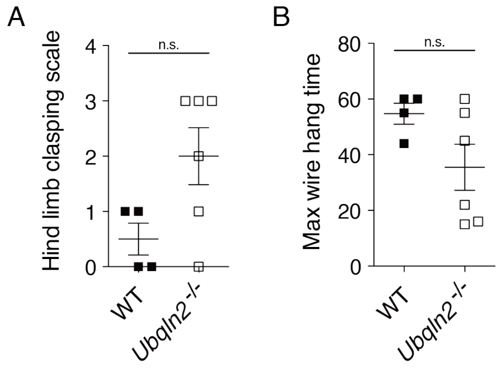

Supplemental Figure 3. Neuromotor testing of young animals.

Animals of approximately 4-5 months of age were tested for hind limb clasping (A) and maximum wire hang time (B) as in Figure 1. n=4-6 animals per genotype, and statistical significance was determined via unpaired, two-tailed Student's T test.

Supplemental Figure 4

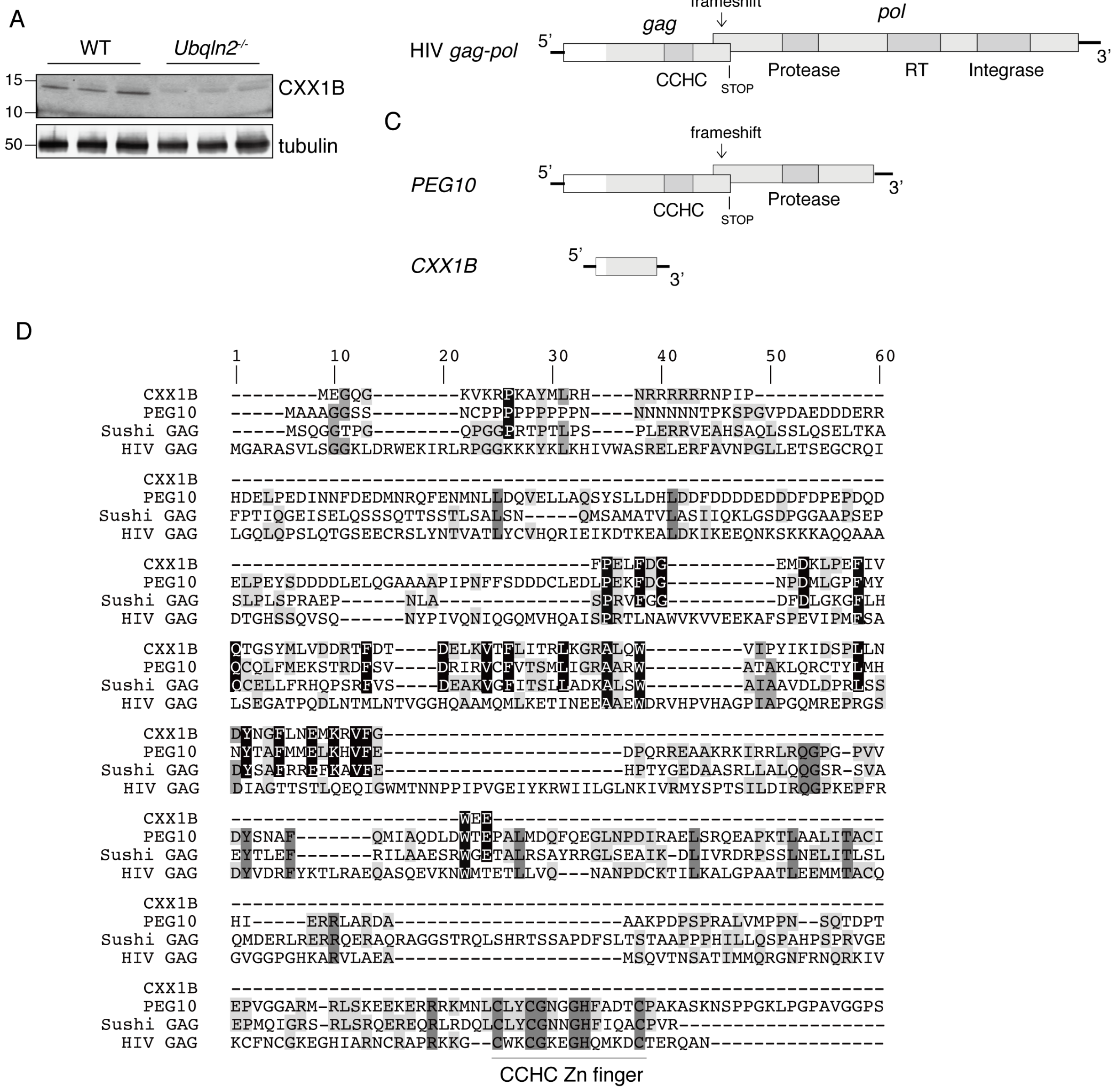

Supplemental Figure 4. Confirmation of proteomic hit CXX1B and comparison to other GAG family proteins.

(A) Proteins from spinal cords of 6-month old WT and *Ubqln2*$^{-/-}$ animals were solubilized in 8 M urea buffer and subjected to Western blotting using antibodies to CXX1B and loading control tubulin. Numbers at left indicate location of molecular weight markers in kDa.

(B) Schematic of a representative *gag*-*pol*-containing retrovirus. The early stop codon is denoted with 'STOP', which is in close proximity to a frameshift-inducing mRNA sequence. Frameshifting bypasses the stop codon, resulting in translation of a second reading frame encoding multiple viral proteins such as reverse transcriptase (RT). Functional domains of the *gag* and *pol* regions (in light grey) are boxed in dark grey.

(C) Schematic of mRNA transcribed from the Ty3/Gypsy retroelement family members *Peg10* and *Cxx1b* identified as UBQLN2 proteomic hits in Figure 2. Top: *Peg10* mRNA organization showing protein domains as dark grey boxes. Bottom: *Cxx1b* mRNA organization with annotated *gag* domain.

(D) Amino acid alignment of CXX1B, murine GAG domain of PEG10, ancestral Sushi-ichi GAG (AAC33525), and HIV GAG (AAA76686). Amino acids with 100% conservation among Ty3/gypsy retroelement family members are highlighted in black. Amino acids conserved among PEG10, Sushi GAG, and HIV GAG, but not CXX1B, are highlighted in dark grey; amino acids conserved among three of four aligned sequences in medium grey; and amino acids conserved among only two aligned sequences in light grey. The CCHC domain found in PEG10 and in Sushi/HIV GAG proteins is noted at bottom. Alignment was performed using MUSCLE algorithm in Geneious with eight iterations.

Supplemental Figure 5

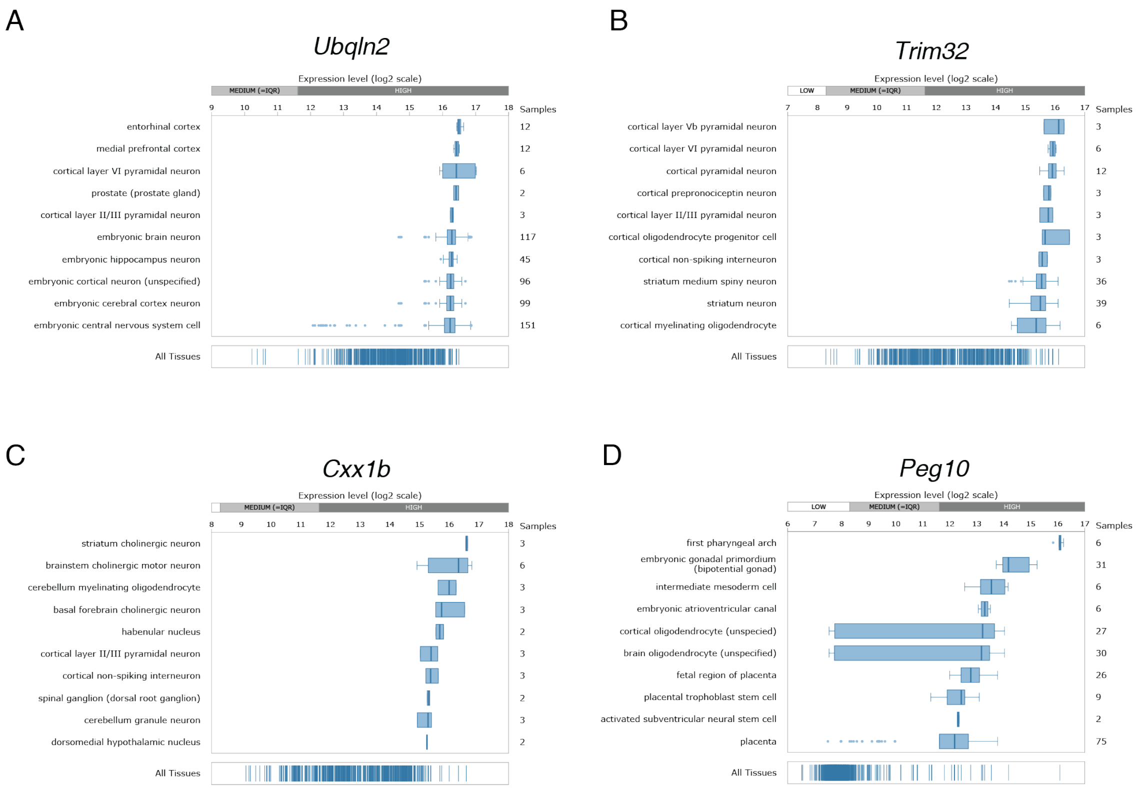

Supplemental Figure 5. Gene expression in multiple mouse tissues.
(A) *Ubqln2*, (B) *Trim32*, and (C) *Cxx1b* mRNA are expressed at relatively high levels in neuronal tissues. (D) *Peg10* is expressed in the brain (see Supplemental Figure 6) but there are non-neuronal cell types with higher levels such as gonad and placenta.
Data are extracted from Genevestigator, a search engine that allows the comparison and analysis of the transcriptional regulation of single genes across multiple species, tissues and conditions.

Supplemental Figure 6

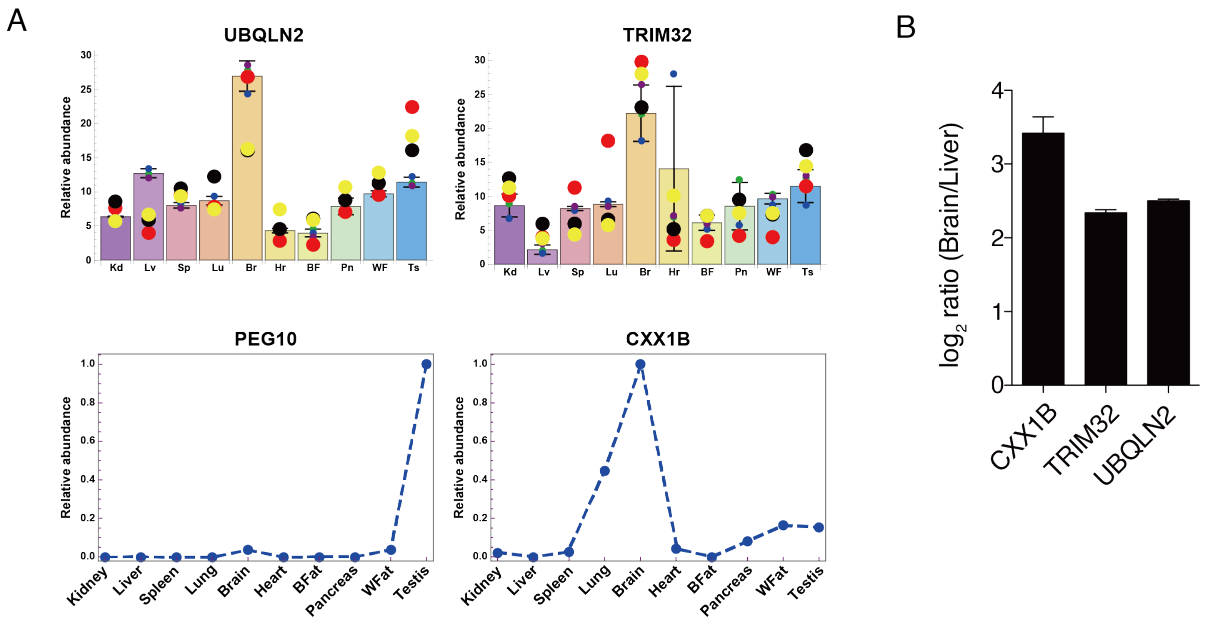

Supplemental Figure 6. Expression levels of specific proteins in multiple mouse tissues.
(A) Expression at the protein level of UBQLN2 and of *Ubqln2*$^{-/-}$ proteomic hits in 10 different tissues of mice. TRIM32, CXX1B and UBQLN2 are most abundant in brain. The two upper graphs represent TMT-MS3 data, the lower graphs label-free quantifications. Kd, Kidney; Lv, Liver; Sp, Spleen; Lu, Lung; Br, Brain; Hr, Heart; BF, Brown Fat; Pn, Pancreas; WF, White fat; Ts, Testis.
(B) Comparison of brain and liver proteomes; CXX1B, TRIM32 and UBQLN2 are more abundant in the brain. PEG10 was not quantified in this dataset. Data extracted from (111).

Supplemental Figure 7

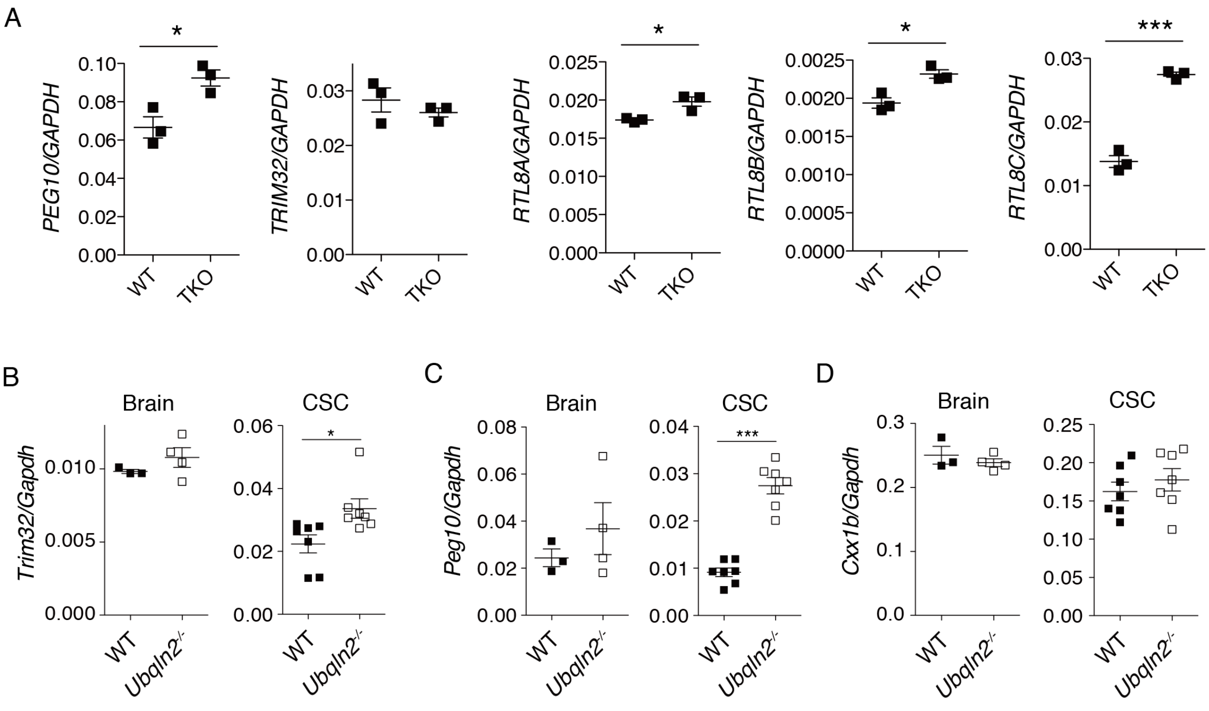

Supplemental Figure 7. mRNA levels of top hits in HEK 293 cells and in mouse tissues.
mRNA abundance of shared proteomic hits from Figures 2 and 3.
(A) mRNA of endogenous human *PEG10*, *TRIM32*, and *RTL8A-C* in WT and TKO HEK 293 cells. n=3 independent experiments.
(B) mRNA of murine *Trim32* (B), *Peg10* (C), and *Cxx1b* (D) as measured by QPCR of brain (left, at approximately one year of age) and cervical spinal cord (CSC, right, at approximately 5-6 months of age) of WT and *Ubqln2*$^{-/-}$ animals. Statistics were determined via unpaired, two-tailed Student's T test. n=3-4 animals per genotype for brain and seven animals per genotype for spinal cord.

Supplemental Figure 8

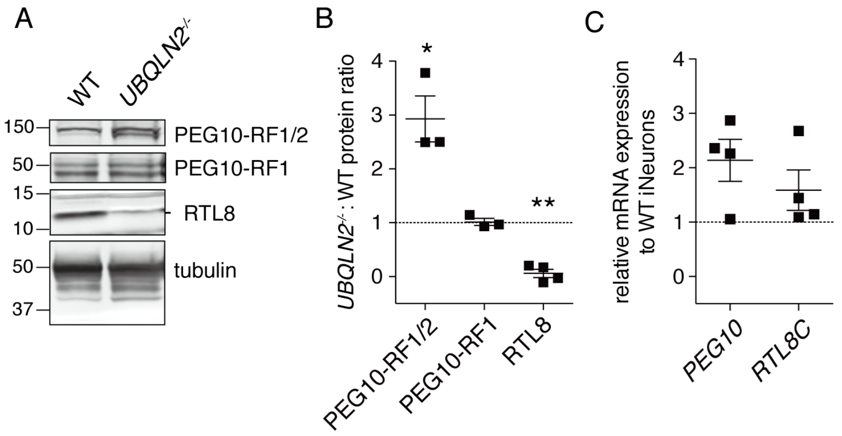

Supplemental Figure 8. Quantifying alteration of top hits in human iNeurons.
(A) Representative Western blot of PEG10-RF1/2, PEG10-RF1, and CXX1B in human induced neurons (iNeurons) after seven days of differentiation from ES cell precursors.
(B) Quantification of the protein abundance of the top hits in *Ubqln2*$^{-/-}$ iNeurons compared to WT controls (normalized to one for each independent differentiation experiment). Protein abundance were first normalized to tubulin levels, then *Ubqln2*$^{-/-}$ iNeuron protein levels were normalized to WT expression levels of the same proteins.
(C) Quantification of mRNA levels of top hits from (A) as measured by QPCR with gene expression normalized to GAPDH. Following GAPDH normalization, *Ubqln2*$^{-/-}$ iNeuron mRNA levels were normalized to WT expression levels of the same genes. Statistical significance was determined via a paired, two-tailed Student's T test.

Supplemental Figure 9

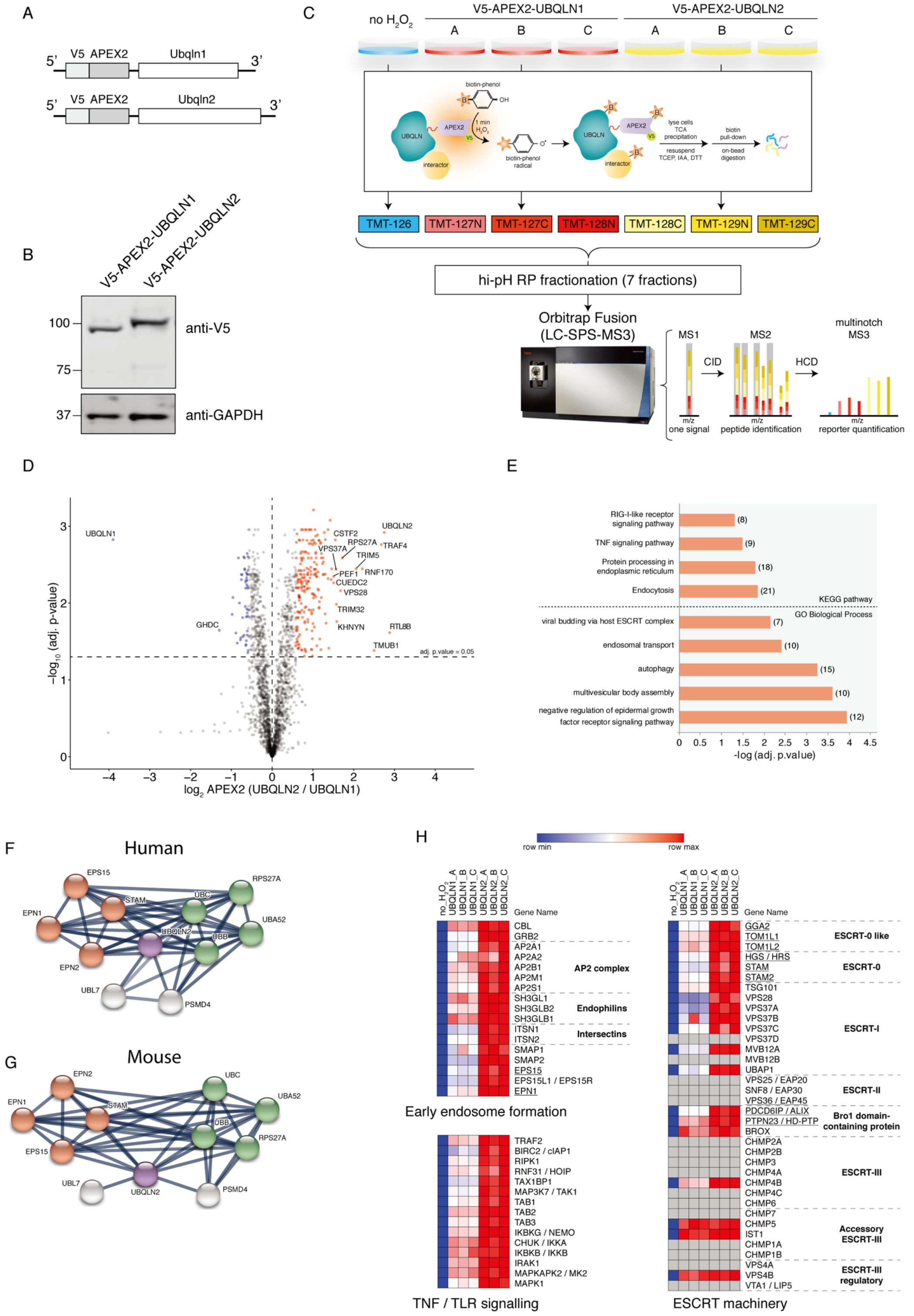

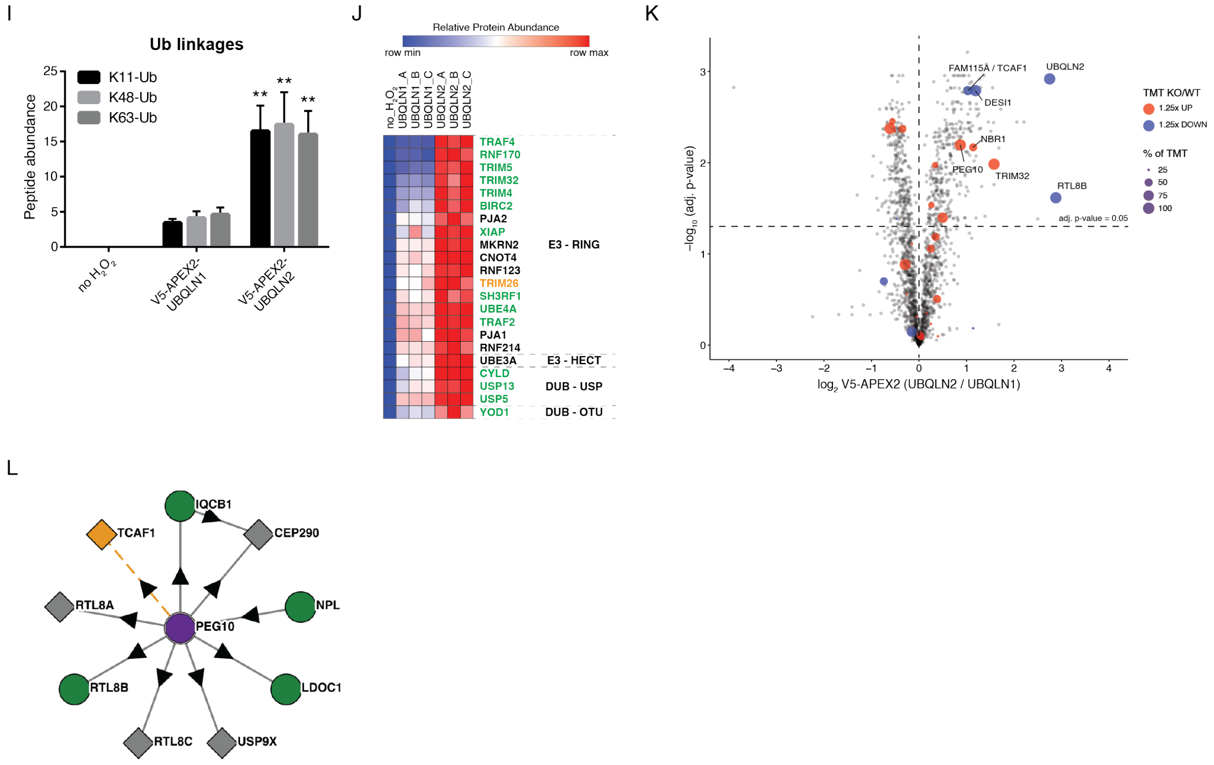

Supplemental Figure 9. Proximity labeling of UBQLN1 and UBQLN2.

(A) Schematic of V5-APEX2-Ubqln fusion constructs used.

(B) Western blot against V5 tag of V5-APEX2-UBQLN1/2 constructs in transiently transfected TKO HEK 293 cells (in the absence of H_2O_2). GAPDH is a loading control.

(C) Schematic representation of the experimental workflow, as detailed in Methods.

(D) Volcano plot with $\log_2$ ratios of mean protein abundances of all 2,323 proteins quantified, with increasing significance on the y-axis (adjusted p-value of 0.05 noted with y-axis dashed line). In red are the 211 proteins with a 1.5-fold or greater enrichment (adjusted p-value < 0.05) in APEX2-UBQLN2. In blue are the 47 proteins with a 1.5-fold or greater enrichment (adjusted p-value < 0.05) in cells expressing APEX2-UBQLN1.

(E) The 211 proteins enriched in APEX2-UBQLN2 samples were used to perform pathway enrichment analysis for gene ontology (GO) biological process terms and KEGG pathways. Processes and complexes implicated in the endocytic pathway are significantly enriched.

(F and G) Interactors of human (F) and mouse (G) UBQLN2, based on the STRING database (a protein-protein interaction database) using interactions with a minimum score of 0.9. Proteins with the 10 highest interaction scores are included. Line thickness indicates the strength of data support (89). In red are proteins involved in endocytosis. In green are the four genes that encode ubiquitin protein.

(H) Heatmaps of proteins involved in endosome formation, TNF/TLR signaling, and ESCRT machinery. For the ESCRT pathway, proteins that recognize polyubiquitin chains containing K63-linkages are underlined.

(I) Ubiquitin linkages quantified by APEX2 mass spectrometry. Each bar represents the mean of each triplicate. Error bars represent standard deviation. Statistical significance was determined with unpaired, two-tailed Student's T test.

(J) Heatmaps of all E3-ligases and deubiquitinating enzymes (DUBs) enriched more than 1.5-fold in APEX2-UBQLN2 samples. In green are proteins with published links to K63-ubiquitination. In orange are those with potential links to K63-ubiquitination.
(K) Volcano plot from panel D that includes only those proteins from the APEX2 study that were also quantified in at least one TMT of *Ubqln2*$^{-/-}$ mice (Figure 2). In red are proteins that were increased by at least 25%; and in blue, proteins that were decreased by at least 25% in *Ubqln2*$^{-/-}$ tissues (KO), with a p-value < 0.05 (Figure 2). The size of each colored dot indicates the percentage of *Ubqln2*$^{-/-}$ tissue-based TMT experiments in which each protein was altered by at least 25%.
(L) Interactome analysis of human PEG10 in HEK 293 cells, adapted from BioPlex (109). The purple circle represents queried protein (PEG10); green circles are bait proteins; gray diamonds are prey proteins; the orange square represents a subthreshold interactor. Arrows indicate bait-to-prey testing relationships. Morpheus (https://software.broadinstitute.org/morpheus) was used to generate heatmaps. See Supplementary Table 4 for all protein quantifications, GO and KEGG enrichment analysis results, and a list of references that link the functions of the proteins listed in panel J with K63-linkages.

Supplemental Figure 10

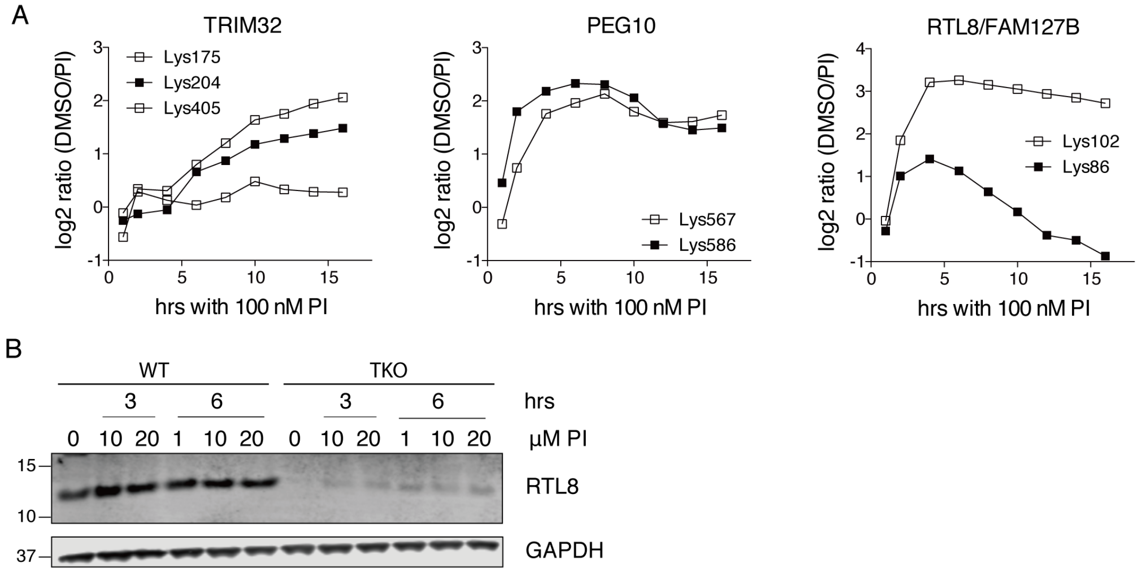

Supplemental Figure 10. Quantifying accumulation of top hits upon inhibition of protein degradation.

(A) Sites of ubiquitin modification (GlyGly sites) were quantified for TRIM32 (left), PEG10 (middle), and RTL8/FAM127B (right) after treatment with proteasome inhibitors (PI, 100 nM bortezomib) in HCT116 cells. Different symbols reflect unique ubiquitinated peptides quantified by GlyGly-TMT analysis. Data are adapted from (111).

(B) WT or TKO HEK cells were incubated with high doses of proteasome inhibitors (PI, bortezomib plus epoxomicin at 1 to 20 µM each) for either three or six hrs, and probed for RTL8 protein levels. GAPDH was used as loading control.

Supplemental Figure 11

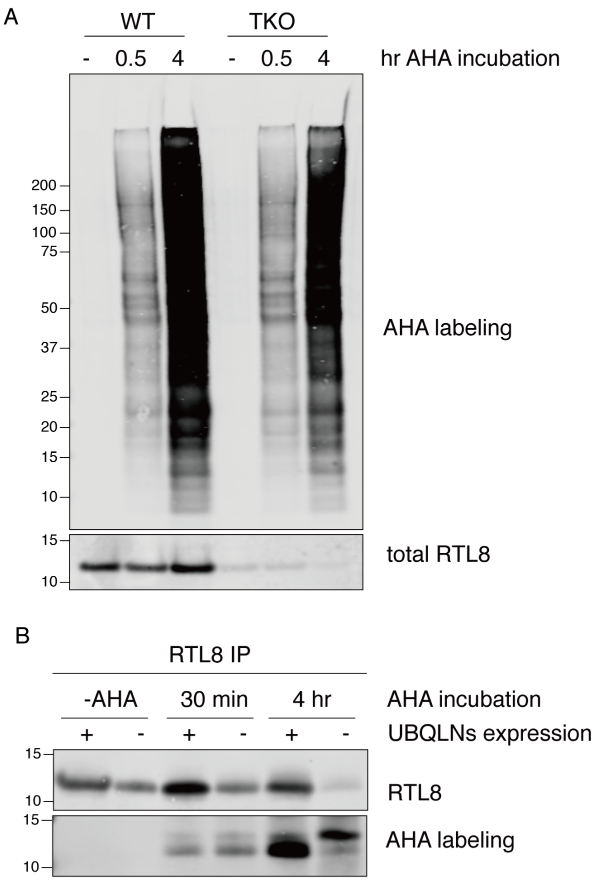

Supplemental Figure 11. The rate of RTL8 synthesis is not reduced in UBQLN-deficient cells.
WT or TKO HEK 293 cells were incubated with the methionine analog AHA in order to label nascent protein. Following AHA incubation, RTL8 was immunoprecipitated to visualize nascent versus total RTL8 protein.
(A) AHA labeling of whole cell lysate. Cells were incubated with regular (-) or AHA-containing media for 30 min or 4 hr following 45 min of methionine starvation. Following lysis, nascent protein was labelled with biotin via click-chemistry and probed with streptavidin. Endogenous total RTL8 levels were also quantified.
(B) AHA labeling of immunoprecipitated RTL8 protein. RTL8 was immunoprecipitated (using anti-RTL8 antibody) from AHA-labelled lysate from 10^7 cells per condition, labelled with biotin via click-chemistry, and the proportion of total RTL8 was determined via streptavidin probe. Shown is one of two representative experiments.

Supplemental Figure 12

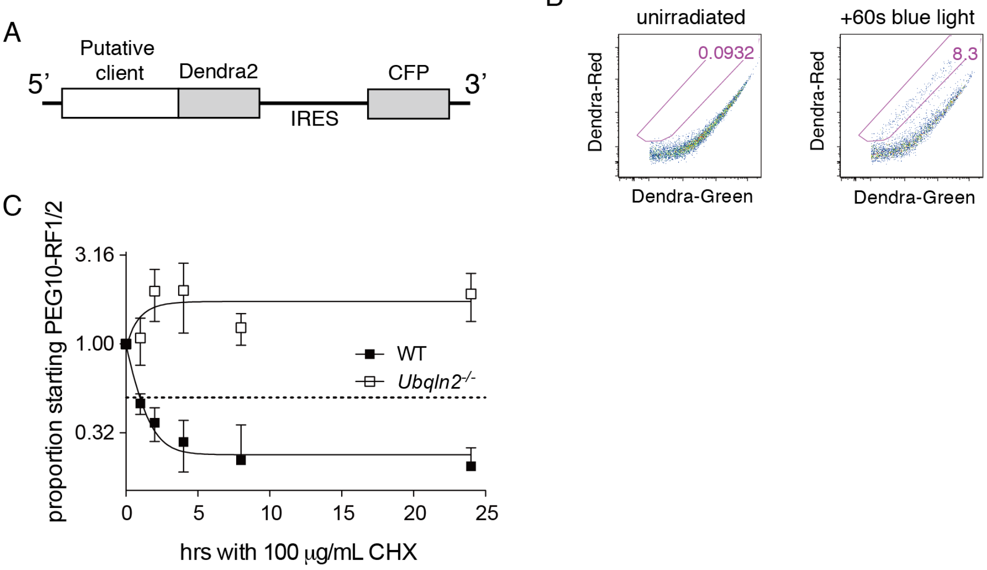

Supplemental Figure 12. Design of Dendra2 constructs and determination of the degradation rate of endogenous full-length PEG10 in human iNeurons.

(A) Design of Dendra2 fusions to putative UBQLN2 client proteins used in Figure 3C-E.

(B) Representative flow cytometry dot plots demonstrating GFP-Dendra2 signal and photoconversion in cells transfected with a control fusion protein of the actin localization sequence fused to Dendra2 (LifeAct-Dendra2). Green Dendra2 fluorescence was observed with Alexa Flour 488 filter, and red Dendra2 fluorescence was observed with PE filter. Cells that become positive for red Dendra2 signal at one hour post-irradiation were gated and the percentage of cells with photoconverted protein is shown in pink.

(C) Degradation rate of endogenous full-length PEG10 protein in human iNeurons. Cells were treated at time zero with 100 μg/mL cycloheximide for 24 hrs to block the synthesis of new protein, and samples were taken at the indicated timepoints for Western blotting. n=4 independent experiments.

Supplemental Figure 13

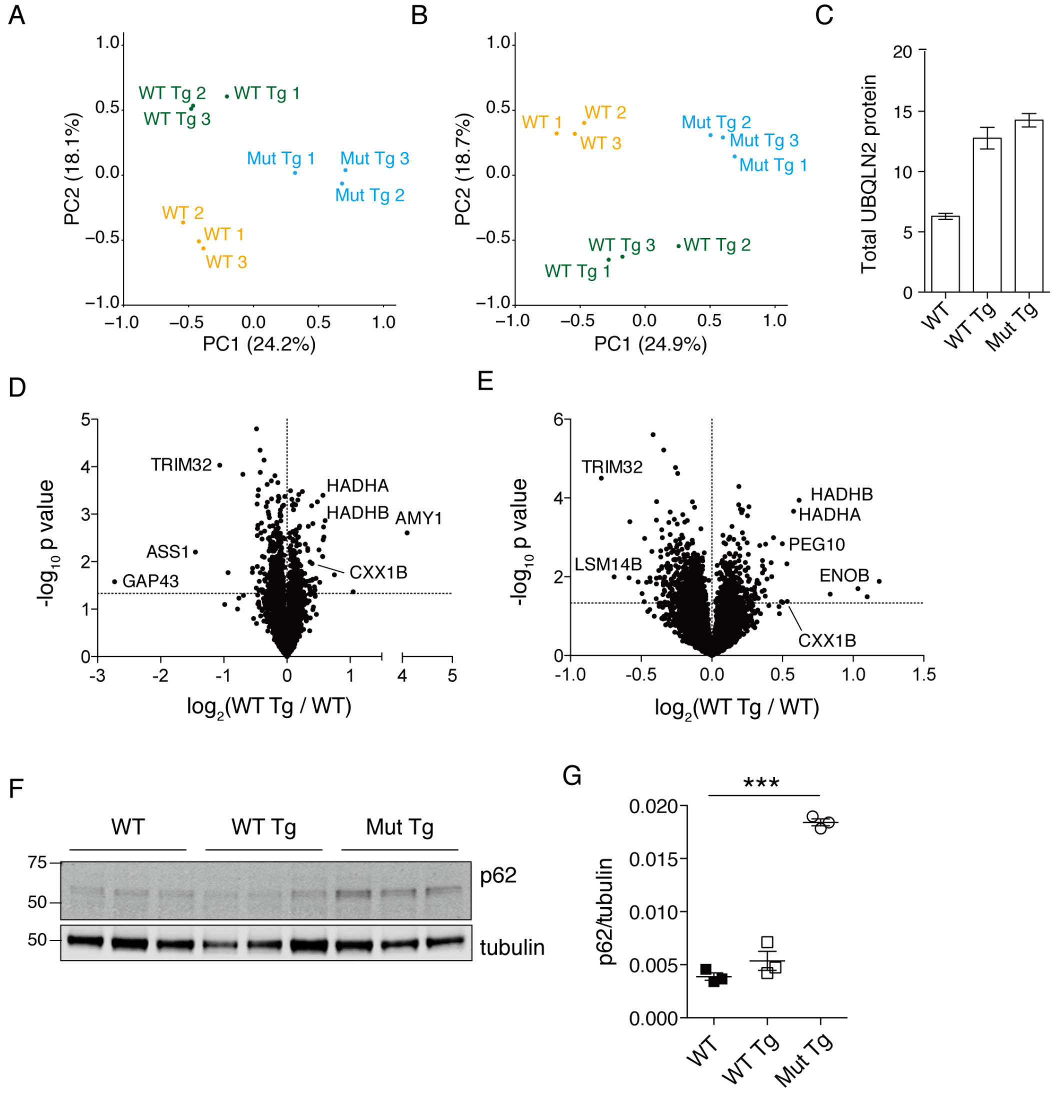

Supplemental Figure 13. Proteomic results from transgenic *Ubqln2* overexpression mice.
(A-B) PCA analysis of transgenic hippocampus (A) and spinal cord (B) samples. Transgene-negative (WT) mice are in yellow, WT transgene-overexpressing mice (WT Tg) in green, and mutant transgene-overexpressing mice (Mut Tg) in blue.
(C) Quantification of total combined mouse and human UBQLN2 in transgenic mice. Two peptides shared between mouse and human UBQLN2 were quantified by TMT 10-plex in hippocampal tissue from WT, WT Tg, and mutant Tg mice.
(D-E) Hippocampus (D) and lumbar spinal cord (E) proteins from transgenic mice expressing WT *UBQLN2* (WT Tg) were compared to those of transgene-negative littermates (WT). n=3 animals per genotype.
(F) Protein samples from *Ubqln2* transgenic mice were prepared as in Figure 5 and Supplemental Figure 4 for Western blotting confirmation of SQSTM1/p62 protein changes.
(G) Quantification of (F) following normalization to tubulin. Statistical significance was determined by using an unpaired, two-tailed Student's T test.

APEX2 Supplementary Discussion

Proximity labeling of UBQLN1 and UBQLN2

We performed a peroxide-catalyzed proximity biotin-labeling (APEX2) experiment with the following objectives: to identify UBQLN2 clients as typically understood–proteins whose degradation is promoted by UBQLN2; to identify possible clients whose degradation is not promoted by UBQLN2; and to identify possible cofactors of UBQLN2. As a quantitative control we used the closely related and widely expressed UBQLN1 protein. UBQLN1 lacks a PXX domain, which in the case of UBQLN2 is the site of the majority of ALS-linked mutations, although the functions of this domain are poorly understood. PXX domain mutations are thought to impair the delivery of client proteins to the proteasome (whether directly or indirectly), to impair Hsc70-UBQLN2 association, and to modulate the self-association of UBQLN2 into aggregates and phase-separated liquid droplets (29, 33, 43, 45). UBQLN1 and UBQLN2 are otherwise highly similar at the amino acid level, with their ubiquitin-recognizing UBA domains for example being ~98% identical. In summary, the APEX2 experiment is designed to detect functions of UBQLN2 that are specific in that they are not shared by UBQLN1, while functions shared with UBQLN1 will be poorly detected if at all as a consequence of normalization.

UBQLN2 and control UBQLN1 constructs were fused to a modified V5-tagged plant peroxidase (V5-APEX2) (86) (Supplemental Figure 9A), and the resulting chimeric proteins were transiently transfected into an HEK 293-derived cell line, TKO HEK 293, carrying a triple knockout of the *UBQLN1, UBQLN2 and UBQLN4* genes (30) (Supplemental Figure 9B). After preincubation in the presence of biotin-phenol, proximity labeling was initiated by the addition of hydrogen peroxide (H_2O_2) for 1 min, after which the cells were lysed and biotin-labelled proteins pulled down with streptavidin beads. A TMT analysis was performed on isolated biotinylated proteins. A control with no H_2O_2 treatment was also included (Supplemental Figure 9C). TMT-MS3 analysis allowed us to quantify 2,323 proteins in total. A direct comparison of the proteins pulled down after APEX2-UBQLN2 labeling versus APEX2-UBQLN1 labeling allowed for the identification of 211 proteins with a 1.5-fold or greater enrichment (adjusted p-value < 0.05) in APEX2-UBQLN2 expressing cells and 47 proteins in cells expressing APEX2-UBQLN1 (Supplemental Figure 9D, red and blue dots respectively).

GO analysis of proteins more abundant in APEX2-UBQLN1 samples showed a significant enrichment for protein translation initiation (Supplemental Table 4). In contrast, GO and KEGG

pathway analysis of proteins more abundant in APEX2-UBQLN2 samples showed a significant enrichment for several processes and complexes related to the endocytic pathway, such as regulation of receptors (e.g., epidermal growth factor receptor [EGFR] and tumor necrosis factor receptor [TNFR]), endosomal transport, multivesicular body assembly, and endosomal sorting complex required for transport (ESCRT) (Supplemental Table 4 and Supplemental Figure 9E).

UBQLNs interact and regulate EGFR levels (87). Previous work published by the Pasterkamp lab identified, among others, several components of the endo/lysosomal trafficking pathway as interactors of UBQLN2 (88). Moreover, many interactors annotated in STRING (89) for human and mouse UBQLN2 are implicated in endocytic processes (CBL, STAM, STAM2, HGS, SH3KBP1/CIN85, EPS15, EPS15L1, EPN1 and EPN2, red nodes in Supplemental Figure 9F and G). These interactors are all involved in receptor tyrosine kinase (RTK) endocytosis, a process which has been extensively studied for EGFR (90, 91). Endocytosis of RTKs is a multistep, ubiquitin-dependent process that follows receptor engagement by cognate ligand and requires the recruitment of many cytosolic components (92). For EGFR, as well as many other RTKs, an early step following ligand binding is the association of the RTK with growth-factor receptor bound-2 (GRB2) and the E3 ubiquitin-protein ligase CBL, which mediates the ubiquitination of the RTK and other components of the machinery. CBL also promotes recruitment of the clathrin coat, SH3KBP1/CIN85, the AP2 adaptor complex, endophilins, epidermal growth factor receptor substrate 15 (EPS15), epidermal growth factor receptor substrate 15-like 1 (EPS15L1/EPS15R), epsins (EPN1 and EPN2), SMAP, and intersectins, among others. As shown in Supplemental Figure 9H, many of these components are enriched in APEX2-UBQLN2 samples. These results corroborate previous observations reported by N'Diaye et al. (81), who described that UBQLN2, and not UBQLN1, specifically interacts with the clathrin-mediated endocytic machinery (EPS15, EPN1 and EPN2) and negatively regulates the endocytosis of G protein-coupled receptors (GPCRs) (81).

For ubiquitinated RTKs to undergo endocytosis and sorting into intraluminal vesicles (ILVs) or multivesicular bodies (MVBs), the ubiquitinated receptor is recognized by the ESCRT machinery, which consists of four major complexes (ESCRTs -0, -I, -II, and -III) and accessory factors that regulate their function. Ubiquitinated receptor is first recognized by HRS, STAM1/2, TSG101 and proteins of the ESCRT II complex. Other components are also capable of binding ubiquitinated receptor and resemble ESCRT-0 subunits; these include TOM1, TOM1L1 and a

family of Arf-dependent clathrin adaptors (GGAs) (93). In our APEX2 analysis, many ESCRT components, especially those of ESCRT-0, ESCRT-I, the accessory Bro1 domain-containing proteins, and the ESCRT-0 like factors, are enriched in cells expressing APEX2-UBQLN2 in comparison to APEX2-UBQLN1 (Supplemental Figure 9H). For more information on how RTKs are internalized, we refer the reader to the following reviews (82, 93, 94, 95).

Based on GO analysis, APEX2-UBQLN2 pull-downs also show enrichment of proteins found in the TNF signaling pathway (Supplemental Figure 9E), where ubiquitination is known to play critical roles through both degradative and non-degradative functions (93). In particular, TNFR-associated proteins (TRAF2, RIPK), and several other components that act downstream, are enriched in APEX2-UBQLN2 when compared to APEX2-UBQLN1 (Supplemental Figure 9H). In addition, TRAF4 was almost undetectable in pull-downs of APEX2-UBQLN1 samples but the most highly enriched protein in cells expressing APEX2-UBQLN2 (Supplemental Figure 9D and 9H). TRAF4 is a RING domain containing ubiquitin ligase that mediates signaling via TNFR as well as the Interleukin-1 receptor (IL1R), Toll-like receptors (TLRs), and the TGFβ receptor. TRAF4 is reported to have roles in cancer and neurodegenerative disease (96, 97). Finally, like RTKs, TNFR also binds ESCRT complexes when bound by ligand and channeled towards degradation (98).

By examining the peptides generated from ubiquitin in our APEX2 study we were also able to quantify three ubiquitin linkages: Lys11, Lys48 and Lys63 (K11, K48, and K63), all of which are more abundant in APEX2-UBQLN2 pull-downs than APEX2-UBQLN1 (Supplementary Figure 9I). The capacity of UBQLN proteins to bind polyubiquitin chains has typically been tested using isolated UBA domains, which do not show a major preference for specific Ub-linkages. However, as recently reported by the Monteiro lab, full-length UBQLN1 binds preferentially to K63-linked ubiquitin chains (25). In our APEX2 experiment, we observed an increase of K63 ubiquitin linkages in APEX2-UBQLN2 pull-downs compared to APEX2-UBQLN1 pull-downs. This is of particular interest due to their importance in TNF and endosomal signaling (83-85, 99).

Of the 18 E3-ligases and 4 DUBs showing a 1.5-fold or greater enrichment in APEX2-UBQLN2 samples, more than half have known functions related to K63 linkages (Supplementary Figure 9J, protein names in green). Among these hits, three TRIM ligases (TRIM4, TRIM5 and TRIM32) are associated with K63-linked ubiquitination and are important in antiviral responses (66, 100, 101). Signal transduction of RLRs (RIG-I-Like Receptors that act primarily as

cytoplasmic sensors for various forms of viral RNA) depends on their ubiquitination state to activate the signaling partner MAVS. For example, TRIM4 modifies RIG-I with K63 chains and CYLD deubiquitinates it (100, 102), whereas conjugates of MAVS are deubiquitinated by YOD1 (103). TRIM4, CYLD, and YOD1 are all enriched in APEX-UBQLN2 samples (Supplementary Figure 9J). TRIM32 catalyzes the formation of K63-linked polyubiquitin chains on STING (STimulator of INterferon Genes, a sensor of cyclic dinucleotides secreted by intracellular bacteria) (66), which enhances downstream signaling by STING. TRIM32 is a known interactor of UBQLN2 (88), and is of particular interest as a novel client of UBQLN2 given its accumulation in mutant mouse neural tissues (Figure 2, 3, 5 and Supplementary Figure 9K). A more detailed list that includes all E3-ligases and DUBs that are more abundant in APEX2-UBQLN2 samples (with references that highlight their connection with K63-linked ubiquitin chains) is included in Supplementary Table 4.

Several ubiquitin receptors that recognize K63 linkages are also enriched in the APEX-UBQLN2 samples. In addition to those implicated in endosomal sorting of receptors (proteins underlined in Supplemental Figure 9H), we identified several linked to the autophagy machinery. NBR1 and Sequestosome/p62 are cargo adaptors that deliver targets modified by K63 ubiquitin chains to autophagosomal machinery (104-106).

In regard to the individual proteins highlighted as clients of UBQLN2 in the main body of this manuscript, we were able to confirm RTL8/CXX1B, TRIM32 and PEG10 were enriched in APEX2-UBQLN2 pull-downs in comparison to APEX2-UBQLN1 (Supplemental Figure 9K). RTL8/CXX1B provides an exceptional case in that UBQLN2 uncharacteristically promotes rather than inhibits its degradation (Figure 2D, Figure 3A, and Supplemental Figure 13D-E), so that UBQLN2 expression in the TKO cells is expected to stabilize RTL8 in the APEX2 experiment. Consequently, we cannot exclude the possibility that the marked enrichment of RTL8/CXX1B in this experiment results from its increased abundance in the TKO cells (although this would nonetheless indicate marked specificity versus UBQLN1).

We also found a number of proteins that specifically interact with UBQLN2 and were significantly altered at the protein level in UBQLN2 mutant neural tissues of mouse (Supplemental Figure 9K), such as DESI1, a desumoylating protease (107) previously reported to work together with UBQLN4 to export ubiquitinated proteins from the nucleus (108). Among this set of proteins, FAM115A/TCAF1 is notable as a previously identified interactor of PEG10 (Supplemental Figure

9L and (109, 110); also FAM115A/TCAF1 with PEG10 have both been identified in UBQLN2 immunoprecipitates (88)). Therefore, RTL8, FAM115 and PEG10 could potentially be part of the same complex.

Altogether, this analysis of UBQLN2 interactors via APEX2 provides a valuable overview of the potential participation of UBQLN2 in endosomal trafficking, TNF signaling, and other processes involving K63-linked ubiquitin chains, some of which have already been suggested in other studies. Clearly, additional experimental work will be necessary to validate these interactions and their precise biological roles, and to determine whether they are relevant to ALS.
