# Supplementary material for: Global proteomics of Ubqln2-based murine models of ALS
Source: J Biol Chem. 2020 Dec 10;296:100153. doi: 10.1074/jbc.RA120.015960 (PMC7873701; doi:10.1074/jbc.RA120.015960)
Supplement: Supplementary file 7 — Table S6 [file mmc7.pdf]

Supplemental Table 1: Antibodies used in study

Immunogen	Clonality	Species	Vendor	Catalog	Usage	Purpose
UBQLN2*	Monoclonal 6H9	Mouse	Novus	NBP2-25164	1:2000	WB
UBQLN4	Polyclonal	Rabbit	Genetex	N2-C2	1:1000	WB
PEG10	Polyclonal	Rabbit	Proteintech	14412-1-AP	1:1000	WB
CXX1b/RTL8	Polyclonal	Rabbit	Proteintech	20282-1-AP	1:1000	WB
TRIM32	Polyclonal	Rabbit	Abcam	ab96612	1:1000	WB
Tubulin	Monoclonal DM1A	Mouse	EMD Millipore	05-829	1:10,000	WB
GAPDH	Monoclonal 6C5	Mouse	Abcam	ab8245	1:10,000	WB
CD68	Monoclonal FA-11	Rat	AbD Serotec	MCA1957T	1:400	IHC
IBA1	Polyclonal	Rabbit	Wako	019-19741	1:15,000	IHC
ChAT	Polyclonal	Goat	EMD Millipore	AB114P	1:1500	IHC
GFAP	Polyclonal	Rabbit	Dako	Z0334	1:200,000	IHC
Ubiquitin	Monoclonal P4D1	Mouse	Santa Cruz Biotechnology	sc-8017	1:1000	WB
Ubiquitin	Polyclonal	Rabbit	Dako	Z0458	1:60,000	IHC
5-HT	Polyclonal	Rabbit	Immunostar	20080	1:30,000	IHC
p62	Monoclonal D6M5X	Rabbit	Cell Signaling Technology	23214	1:1000	WB
HA	Monoclonal HA-7	Mouse	Sigma	H3663	1:500	WB
LC3B	Polyclonal	Rabbit	Novus	NB100-220	1:5,000	WB
mouse IgG	Polyclonal	Goat	Licor (IR dye 680)	926-68070	1:10,000	WB
rabbit IgG	Polyclonal	Goat	Licor (IR dye 800)	926-32211	1:10,000	WB

*Also recognizes UBQLN1 protein
